# Spatiotemporal Trends of Colorectal Cancer Mortality Due to Low Physical Activity and High Body Mass Index From 1990 to 2019: A Global, Regional and National Analysis

**DOI:** 10.3389/fmed.2021.800426

**Published:** 2022-01-10

**Authors:** Jinyu Man, Tongchao Zhang, Xiaolin Yin, Hui Chen, Yuan Zhang, Xuening Zhang, Jiaqi Chen, Xiaorong Yang, Ming Lu

**Affiliations:** ^1^Department of Epidemiology and Health Statistics, School of Public Health, Cheeloo College of Medicine, Shandong University, Jinan, China; ^2^Clinical Epidemiology Unit, Qilu Hospital of Shandong University, Jinan, China; ^3^Clinical Research Center of Shandong University, Qilu Hospital, Cheeloo College of Medicine, Shandong University, Jinan, China; ^4^Department of Gastroenterology, Qilu Hospital, Cheeloo College of Medicine, Shandong University, Jinan, China

**Keywords:** colorectal cancer, high BMI, low physical activity, temporal trend, global disease burden

## Abstract

**Background:** Understanding the spatiotemporal trends of colorectal cancer (CRC) deaths caused by low physical activity (LPA) and high body mass index (BMI) is essential for the prevention and control of CRC. We assessed patterns of LPA and high BMI-induced CRC deaths from 1990 to 2019 at global, regional, and national levels.

**Methods:** Data on CRC deaths due to LPA and high BMI was downloaded from the Global Burden of Disease 2019 Study. We calculated estimated annual percentage change (EAPC) to quantify spatiotemporal trends in the CRC age-standardized mortality rate (ASMR) due to LPA and high BMI.

**Results:** In 2019, CRC deaths due to LPA and high BMI were estimated as 58.66 thousand and 85.88 thousand, and the corresponding ASMRs were 0.77/100,000 and 1.07/100,000, with EAPCs of−0.39 [95% confidence interval (CI):−0.49,−0.29] and 0.64[95% CI: 0.57, 0.71] from 1990 to 2019 respectively. Since 1990, the ASMR of CRC attributable to LPA and high BMI has been on the rise in many geographic regions, especially in low middle and middle sociodemographic index (SDI) regions. Thirteen countries had a significant downward trend in CRC ASMR attributed to LPA, with EAPCs < −1. And, only 4 countries had a significant downward trend in CRC ASMR attributable to high BMI, with EAPCs < −1. Countries with a higher baseline burden in 1990 and a higher SDI in 2019 had a faster decline in ASMR due to high BMI and LPA.

**Conclusions:** The burden of CRC caused by LPA and high BMI is on the rise in many countries. Countries should adopt a series of measures to control the local prevalence of obesity and LPA in order to reduce disease burden, including CRC.

## Introduction

Colorectal cancer (CRC) is the third most common cancer and the second leading cause of death from cancer in the world (1). In 2020, there were more than 1.9 million new cases of CRC and more than 930,000 deaths of CRC in the world (2, 3). The incidence and mortality of CRC in many middle-income and low-income countries are rising rapidly, and while the incidence and mortality of CRC in highly developed countries tend to stabilize or decline after the peak (1, 4). The complex disease situation complicates the formulation of global CRC prevention and control strategies.

Many prospective cohort studies and meta-analyses had confirmed that some changeable lifestyle factors, such as smoking (5), drinking (6), low physical activity (LPA) (7, 8), high body mass index (BMI) (9–11), and dietary (12, 13), were associated with the risk of CRC. In 2019, The Global Burden of Disease (GBD) 2017 Colorectal Cancer Collaborators explored the incidence, mortality, and disability of CRC in 195 countries and territories around the world from 1990 to 2017, and the proportion of CRC disability-adjusted life years (DALYs) attributable to risk factors (smoking, drinking, LPA, high BMI, high fasting plasma glucose and dietary) in 2017, which provided a solid foundation for the prevention and control of CRC (14). Further, Deng et al. explored the geographic and temporal trends of CRC disease burden related to various dietary factors, and found that from 1990 to 2019, CRC deaths and DALYs caused by dietary factors increased by half, and interventions for alterable dietary risk factors would reduce CRC deaths and DALYs by 32 and 34%, respectively (15). In recent years, the prevalence of smoking (16) and drinking (17) has shown a downward trend or has changed little globally, while the prevalence of high BMI (18) and LPA (19) has shown an upward trend in some regions. Understanding the geographic and temporal trends and its influencing factors of CRC disease burden caused by LPA and high BMI will make the formulation of CRC prevention and control policies more targeted. However, at present, almost no research had been conducted on these topics, and the geographic and temporal trends and its influencing factors of the CRC disease burden due to LPA and high BMI were still unknown.

GBD Study 2019 systematically assesses and updates the disease burden of CRC and some influencing factors in 204 countries and territories, which provides a rare opportunity to carry out global, regional and national research on geographic and temporal trends and its related influencing factors of CRC disease burden attributed to LPA and high BMI. In our study, we estimated the geographic and temporal trends of CRC disease burden due to LPA and high BMI by gender and age groups at global, regional and national levels, and further discussed influencing factors of temporal trends. Our research will provide a basis for policy formulation and the allocation of medical resources, and contribute to reducing the CRC disease burden in the population.

## Materials and Methods

### Data Collection

We obtained annual CRC deaths and age-standardized mortality rates (ASMRs) due to LPA and high BMI by sex, country, and 16 age categories (5-year groups within the ages of 20–94 years and ≥95 years) from 1990 to 2019 from the GBD 2019 study (http://ghdx.healthdata.org/gbd-results-tool). Data of 204 countries and territories was collected. Relevant data were reported in numbers and 95% uncertainty intervals (UIs), which were determined by 2.5% and 97.5% of the ordered 1,000 estimates. Based on the sociodemographic index (SDI), which is a composite index and was calculated by aggregating the education level, fertility rate, and income per capita, we further categorized the countries and territories into five regions, namely, low, low-middle, middle, high-middle, and high SDI regions (14, 20). Based on a geographic hierarchy, all 204 countries and territories were separated into 21 GBD regions and then grouped into 7 super GBD regions.

### Definitions of CRC and High BMI and LPA Exposure

All ICD10 and ICD9 codes related to colon cancer and rectal cancer (C18-C19.0, C20, C21-C21.8, Z12.1-Z12.13, Z85.03-Z85.048, Z86. 010 and 153- 154.9, 209.1-209.17, V10.05-V10.06, V76.41, V76.5-V76.52) were included in the CRC mortality estimates. The LPA for adults older than 25 years old was defined as < 3,000 MET-min/week. MET is defined as the oxygen uptake in ml/kg/min. One MET is equal to the oxygen consumption of sitting quietly, which is about 3.5 ml/kg/min. A high BMI for adults over 20 years old was defined as a BMI > 25 kg/m^2^.

### Estimation of CRC Disease Burden Due to LPA and High BMI

The disease burden of CRC caused by LPA and high BMI is estimated by population attributable fraction (PAF), which represents the percentage of risk that would be reduced in a given year if the past exposure to risk factors was reduced to the ideal exposure scenario. The formula of PAF was:


$$\begin{array}{l} PAF=\frac{\sum_{x\;=\;1}^{n}RR\left(x\right)P\left(x\right)-1}{\sum_{x\;=\;1}^{n}RR\left(x\right)P\left(x\right)} \end{array}$$

where *P*(*x*) is the proportion of population exposed to LPA or high BMI at level *x* in the target population and *RR*(*x*) is the relative risk of LPA or high BMI level *x*. LPA exposure data is extracted from surveys of general adults in which random sampling was used. These surveys collected self-reported physical activity in various areas of life (leisure/entertainment, work/family, and transportation). The high BMI exposure data were extracted from the systematic literature review, survey reports and survey microdata. The relative risks in LPA and high BMI for CRC were obtained from pooled studies or published meta-analyses (14, 21).

### Statistical Analyses

We calculated the widely used estimated annual percentage change (EAPC) to measure the time trends of ASMR by sex, age groups, and regions from 1990 to 2019. We put ASMR into the model “ln (ASMR) = α + β^*^ calendar year + ε” and calculated EAPC from the formula 100 × (exp(β)-1). The 95% confidence interval (CI) of EAPC was also generated by this model. If both the EAPC estimate and the lower limit of its 95% CI are > 0, the ASMR is considered to be rising. Conversely, if the EAPC estimate and the higher limit of its 95% CI are < 0, the ASMR is considered to be on a downward trend. In order to explore the influencing factor of EAPC, we used Spearman rank correlation to evaluate the relationship between EAPCs of ASMRs attributed to LPA and high BMI and baseline burden in 1990 and SDI in 2019 in 204 countries and territories considering the non-normal distribution. ASMR attributed to LPA and high BMI in 1990 reflects the baseline disease burden, and the SDI in 2019 reflects the level and availability of healthcare in 204 countries and territories (22–25). We performed all analyses using R (version 4.0.3; https://www.R-project.org/). A *P*-value lower than 0.05 on both sides was considered statistically significant.

## Results

### Estimates and Variation of CRC Deaths Due to LPA and High BMI Across Regions

**Figure 1:**
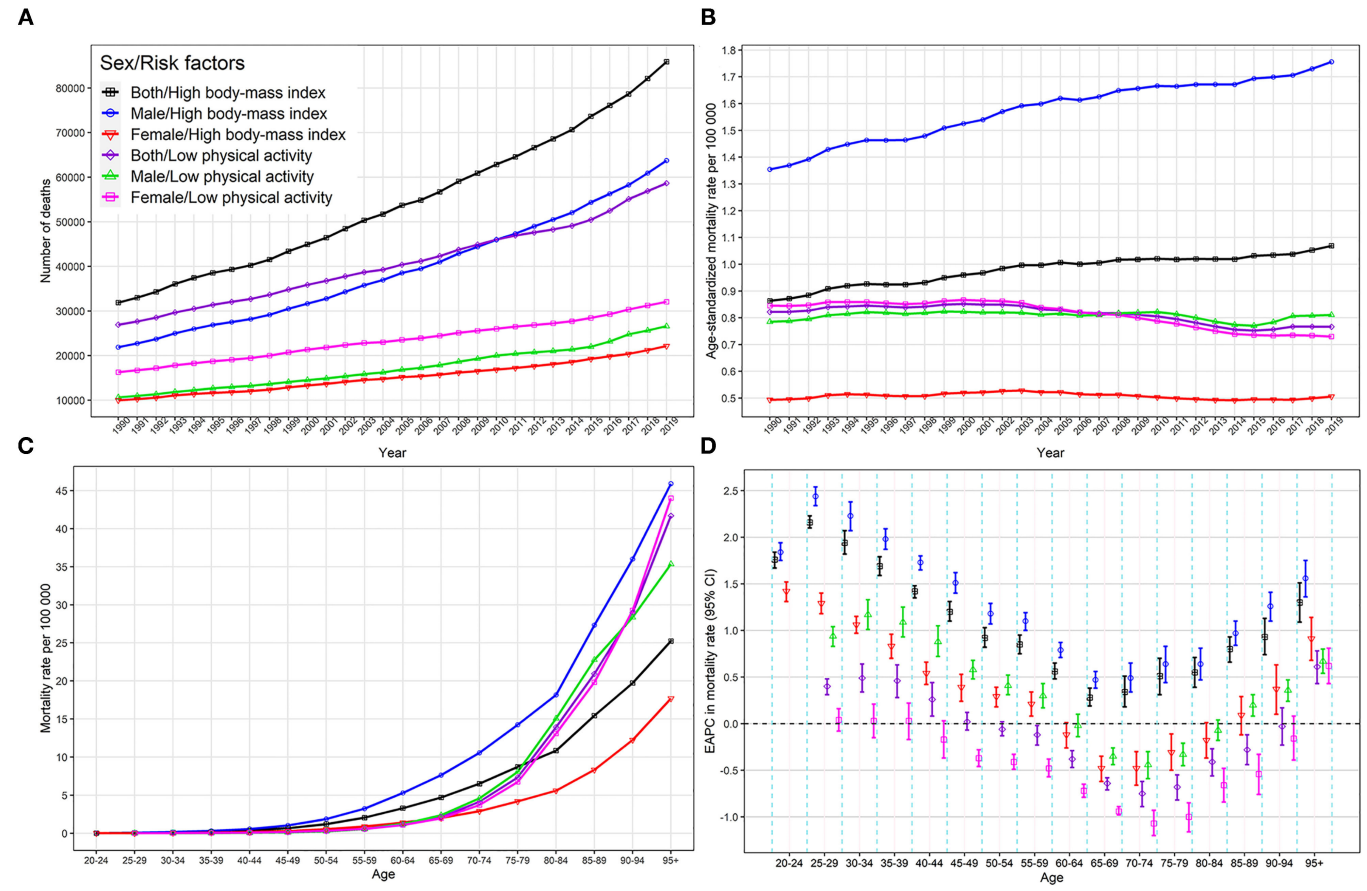
Global colorectal cancer deaths and mortality rates attributable to high BMI and low physical activity. **(A)** global colorectal cancer deaths, 1990–2019; **(B)** global colorectal cancer ASMR, 1990–2019; **(C)** global colorectal cancer mortality rate by age group, 2019; **(D)** EAPC in global colorectal cancer mortality rate by age group, 1990–2019. ASMR, age-standardized mortality rate; EAPC, estimated annual percentage change; BMI, body mass index.

Of all CRC-related deaths, 58.66 thousand (95% UI 16.87–112.15) and 85.88 thousand (95% UI 46.85–136.52) were attributed to LPA and high BMI, with ASMR of 0.77 (95% UI 0.22–1.47) and 1.07 (95% UI 0.58–1.70), respectively (Table 1). The CRC deaths caused by LPA was higher in females than in males, however, the corresponding ASMR was higher in males than that in females, with ASMR of 0.73 (95% UI 0.23–1.35) and 0.81 (95% UI 0.20–1.60) for females and males, respectively. In males, the number and ASMR of CRC-related deaths due to high BMI were approximately three times that in females (Figures 1A,B; Table 1). The mortality rate due to LPA increased after 75 years of age sharply. The mortality rate of CRC caused by high BMI rose significantly after the age of 80, and it rose more rapidly in males (Figure 1C).

**Table 1:** Death cases and ASMR of colorectal cancer due to low physical activity and high BMI in 1990 and 2019 and the temporal trends from 1990 to 2019.

**Characteristics**	**1990**				**2019**				**EAPC (1990–2019)**	
	**High BMI**		**Low activity**		**High BMI**		**Low activity**		**High BMI**	**Low activity**
	**Death cases**	**ASMR**	**Death cases**	**ASMR**	**Death cases**	**ASMR**	**Death cases**	**ASMR**		
**Global**	31.90 (15.67, 53.51)	0.86 (0.42, 1.45)	26.93 (6.79, 52.68)	0.82 (0.22, 1.59)	85.88 (46.85, 136.52)	1.07 (0.58, 1.70)	58.66 (16.87, 112.15)	0.77 (0.22, 1.47)	0.64 (0.57, 0.71)	−0.39 (-0.49, −0.29)
**Sex**										
Males	21.90 (10.51, 36.6)	1.35 (0.64, 2.30)	10.64 (2.21, 21.72)	0.79 (0.17, 1.58)	63.73 (33.85, 102.03)	1.76 (0.93, 2.82)	26.59 (6.38, 52.43)	0.81 (0.20, 1.60)	0.83 (0.76, 0.90)	−0.05 (-0.13, 0.03)
Females	10.00 (4.22, 18.78)	0.49 (0.21, 0.93)	16.29 (4.50, 30.39)	0.85 (0.24, 1.56)	22.15 (9.89, 39.93)	0.51 (0.23, 0.91)	32.07 (10.29, 59.14)	0.73 (0.23, 1.35)	−0.08 (-0.17, 0.01)	−0.64 (-0.76, −0.52)
**SDI region**										
High	16.35 (8.20, 26.81)	1.56 (0.78, 2.56)	14.10 (3.35, 27.88)	1.32 (0.32, 2.62)	30.25 (16.53, 46.90)	1.57 (0.87, 2.42)	22.07 (5.66, 42.39)	1.02 (0.25, 1.98)	−0.18 (-0.30, −0.06)	−1.21 (-1.36, −1.05)
High-middle	11.56 (5.94, 18.77)	1.11 (0.57, 1.82)	7.83 (2.12, 15.05)	0.84 (0.23, 1.61)	30.30 (16.71, 47.26)	1.49 (0.82, 2.33)	18.09 (5.49, 34.06)	0.91 (0.28, 1.71)	0.92 (0.81, 1.03)	0.22 (0.11, 0.35)
Middle	2.80 (1.11, 5.53)	0.28 (0.11, 0.56)	3.11 (0.82, 6.14)	0.40 (0.11, 0.76)	18.01 (9.04, 30.08)	0.74 (0.37, 1.25)	12.13 (3.35, 23.5)	0.58 (0.17, 1.11)	3.58 (3.46, 3.70)	1.19 (1.08, 1.29)
Low-middle	0.84 (0.28, 1.79)	0.15 (0.05, 0.32)	1.44 (0.42, 2.76)	0.32 (0.10, 0.59)	5.76 (2.89, 9.63)	0.43 (0.22, 0.73)	5.15 (1.69, 9.57)	0.45 (0.15, 0.84)	3.84 (3.79, 3.89)	1.33 (1.27, 1.38)
Low	0.33 (0.11, 0.71)	0.15 (0.05, 0.32)	0.44 (0.11, 0.88)	0.24 (0.07, 0.48)	1.50 (0.73, 2.60)	0.30 (0.14, 0.53)	1.17 (0.34, 2.28)	0.30 (0.09, 0.57)	2.68 (2.52, 2.85)	1.01 (0.93, 1.09)
**GBD region**										
High-income Asia Pacific	1.23 (0.38, 2.56)	0.63 (0.19, 1.31)	1.98 (0.37, 4.09)	1.09 (0.22, 2.19)	3.12 (1.10, 6.17)	0.67 (0.24, 1.30)	5.55 (1.26, 11)	0.98 (0.20, 1.97)	−0.02 (-0.14, 0.1)	−0.45 (−0.51, −0.40)
High-income North America	6.25 (3.16, 9.80)	1.77 (0.90, 2.77)	4.13 (0.97, 8.20)	1.11 (0.26, 2.21)	12.04 (6.91, 17.46)	1.92 (1.11, 2.77)	4.35 (0.95, 8.52)	0.65 (0.14, 1.27)	0.08 (-0.03, 0.18)	−2.16 (−2.44, −1.88)
Western Europe	9.79 (4.92, 15.94)	1.67 (0.85, 2.72)	9.86 (2.45, 18.75)	1.63 (0.41, 3.13)	16.31 (8.59, 25.69)	1.70 (0.90, 2.64)	14.72 (4.05, 27.6)	1.37 (0.36, 2.56)	−0.18 (-0.40, 0.03)	−0.98 (-1.25, −0.72)
Australasia	0.49 (0.26, 0.78)	2.13 (1.13, 3.34)	0.39 (0.09, 0.76)	1.70 (0.39, 3.28)	0.96 (0.54, 1.44)	1.91 (1.09, 2.83)	0.79 (0.21, 1.44)	1.46 (0.37, 2.70)	−0.72 (-0.84, −0.59)	−0.85 (-0.95, −0.75)
Southern Latin America	0.57 (0.26, 1.00)	1.27 (0.57, 2.21)	0.12 (0.03, 0.29)	0.28 (0.07, 0.68)	1.72 (0.90, 2.68)	2.05 (1.07, 3.18)	0.36 (0.09, 0.78)	0.42 (0.11, 0.91)	1.57 (1.35, 1.79)	1.65 (1.46, 1.84)
Andean Latin America	0.10 (0.05, 0.17)	0.48 (0.22, 0.84)	0.04 (0.01, 0.10)	0.25 (0.05, 0.55)	0.55 (0.29, 0.90)	1.00 (0.52, 1.63)	0.25 (0.05, 0.51)	0.47 (0.10, 0.95)	2.92 (2.72, 3.11)	2.43 (2.25, 2.62)
Tropical Latin America	0.55 (0.26, 0.94)	0.63 (0.30, 1.10)	0.96 (0.31, 1.64)	1.21 (0.44, 2.02)	2.92 (1.68, 4.39)	1.22 (0.70, 1.84)	3.45 (1.43, 5.63)	1.47 (0.62, 2.39)	2.46 (2.18, 2.74)	0.83 (0.65, 1.00)
Central Latin America	0.39 (0.19, 0.65)	0.48 (0.23, 0.81)	0.16 (0.04, 0.36)	0.23 (0.05, 0.51)	2.23 (1.19, 3.51)	0.95 (0.51, 1.50)	0.77 (0.16, 1.61)	0.34 (0.07, 0.72)	2.36 (2.27, 2.45)	1.17 (1.04, 1.31)
Caribbean	0.21 (0.11, 0.35)	0.83 (0.42, 1.37)	0.28 (0.08, 0.51)	1.16 (0.36, 2.08)	0.73 (0.40, 1.14)	1.41 (0.77, 2.21)	0.79 (0.26, 1.38)	1.53 (0.50, 2.66)	2.08 (1.98, 2.19)	1.03 (0.97, 1.08)
Eastern Europe	4.25 (2.37, 6.56)	1.51 (0.84, 2.34)	1.43 (0.42, 2.97)	0.54 (0.16, 1.10)	7.34 (4.32, 10.89)	2.11 (1.25, 3.13)	2.39 (0.74, 4.69)	0.67 (0.21, 1.32)	0.89 (0.69, 1.09)	0.89 (0.73, 1.05)
Central Europe	3.11 (1.77, 4.74)	2.12 (1.20, 3.23)	1.10 (0.32, 2.19)	0.80 (0.24, 1.61)	6.78 (3.94, 10.16)	3.12 (1.81, 4.66)	2.25 (0.66, 4.48)	0.99 (0.29, 1.97)	1.37 (1.27, 1.48)	0.66 (0.56, 0.77)
Central Asia	0.39 (0.21, 0.62)	0.83 (0.43, 1.32)	0.14 (0.04, 0.28)	0.32 (0.10, 0.66)	0.79 (0.45, 1.19)	1.12 (0.63, 1.69)	0.25 (0.07, 0.49)	0.46 (0.14, 0.89)	1.34 (1.05, 1.63)	1.53 (1.35, 1.70)
North Africa and Middle East	1.03 (0.53, 1.74)	0.62 (0.31, 1.04)	1.09 (0.33, 1.97)	0.77 (0.26, 1.36)	4.84 (2.79, 7.15)	1.17 (0.67, 1.74)	3.82 (1.32, 6.59)	1.03 (0.39, 1.75)	2.39 (2.17, 2.61)	1.20 (1.02, 1.39)
South Asia	0.48 (0.15, 1.06)	0.09 (0.03, 0.20)	1.13 (0.32, 2.19)	0.28 (0.08, 0.54)	4.11 (2.04, 6.88)	0.30 (0.15, 0.51)	4.24 (1.41, 7.98)	0.38 (0.13, 0.71)	4.12 (3.94, 4.29)	1.26 (1.08, 1.45)
Southeast Asia	0.51 (0.17, 1.09)	0.20 (0.06, 0.43)	0.68 (0.16, 1.41)	0.35 (0.08, 0.71)	4.16 (2.06, 7.12)	0.68 (0.34, 1.18)	2.98 (0.72, 5.94)	0.60 (0.15, 1.20)	4.28 (4.20, 4.36)	1.72 (1.62, 1.82)
East Asia	1.96 (0.45, 4.65)	0.23 (0.05, 0.55)	3.00 (0.77, 6.1)	0.47 (0.13, 0.95)	14.91 (5.81, 27.94)	0.73 (0.28, 1.39)	10.54 (2.75, 21.24)	0.60 (0.16, 1.19)	4.53 (4.24, 4.81)	0.66 (0.50, 0.82)
Oceania	0.01 (0.01, 0.02)	0.43 (0.18, 0.78)	0.01 (<0.01, 0.02)	0.45 (0.13, 0.86)	0.04 (0.02, 0.07)	0.58 (0.27, 1.01)	0.03 (0.01, 0.05)	0.54 (0.16, 1.02)	0.86 (0.78, 0.95)	0.57 (0.52, 0.62)
Western Sub-Saharan Africa	0.18 (0.07, 0.35)	0.21 (0.08, 0.43)	0.18 (0.05, 0.37)	0.25 (0.07, 0.53)	0.91 (0.47, 1.48)	0.52 (0.27, 0.86)	0.52 (0.13, 1.04)	0.36 (0.10, 0.72)	3.27 (3.18, 3.36)	1.38 (1.30, 1.47)
Eastern Sub-Saharan Africa	0.12 (0.04, 0.25)	0.16 (0.05, 0.35)	0.06 (0.02, 0.15)	0.10 (0.03, 0.25)	0.63 (0.31, 1.09)	0.39 (0.19, 0.69)	0.16 (0.05, 0.41)	0.13 (0.04, 0.31)	3.39 (3.16, 3.63)	1.00 (0.93, 1.07)
Central Sub-Saharan Africa	0.06 (0.02, 0.11)	0.27 (0.11, 0.53)	0.05 (0.01, 0.11)	0.33 (0.09, 0.66)	0.17 (0.08, 0.31)	0.34 (0.16, 0.62)	0.14 (0.04, 0.30)	0.37 (0.10, 0.76)	0.38 (-0.24, 1.00)	0.31 (0.03, 0.59)
Southern Sub-Saharan Africa	0.19 (0.10, 0.31)	0.72 (0.37, 1.21)	0.14 (0.04, 0.28)	0.60 (0.18, 1.20)	0.62 (0.37, 0.94)	1.17 (0.70, 1.76)	0.33 (0.09, 0.63)	0.70 (0.20, 1.34)	1.91 (1.67, 2.14)	0.83 (0.46, 1.21)

*ASMR, age-standardized mortality rate; EAPC, estimated annual percentage change; BMI, body mass index*.

In 2019, the CRC deaths due to LPA were highest in high SDI region [22.07 (95 % UI 5.66–42.39) ^*^10^3^], and followed by high-middle SDI region [18.09 (95 % UI 5.49–34.06) ^*^10^3^]. The highest number of deaths related to CRC caused by high BMI were observed in high and high-middle SDI regions (more than 30,000 cases), with ASMR of 1.57(95% UI 0.87–2.42) and 1.49 (95% UI 0.82–2.33), respectively (Table 1). In high and high-middle SDI regions, people aged 65 and over accounted for the vast majority of CRC-related deaths caused by LPA and high BMI (Supplementary Figure 1). For GBD regions, the largest number of deaths related to CRC caused by LPA and high BMI was found in western Europe, followed by East Asia, and the highest ASMRs of CRC-related deaths due to LPA and high BMI were found in the Caribbean [1.53 (95 % UI 0.50–2.66)] and Central Europe [3.12 (95 % UI 1.81–4.66)], respectively (Table 1).

**Figure 2:**
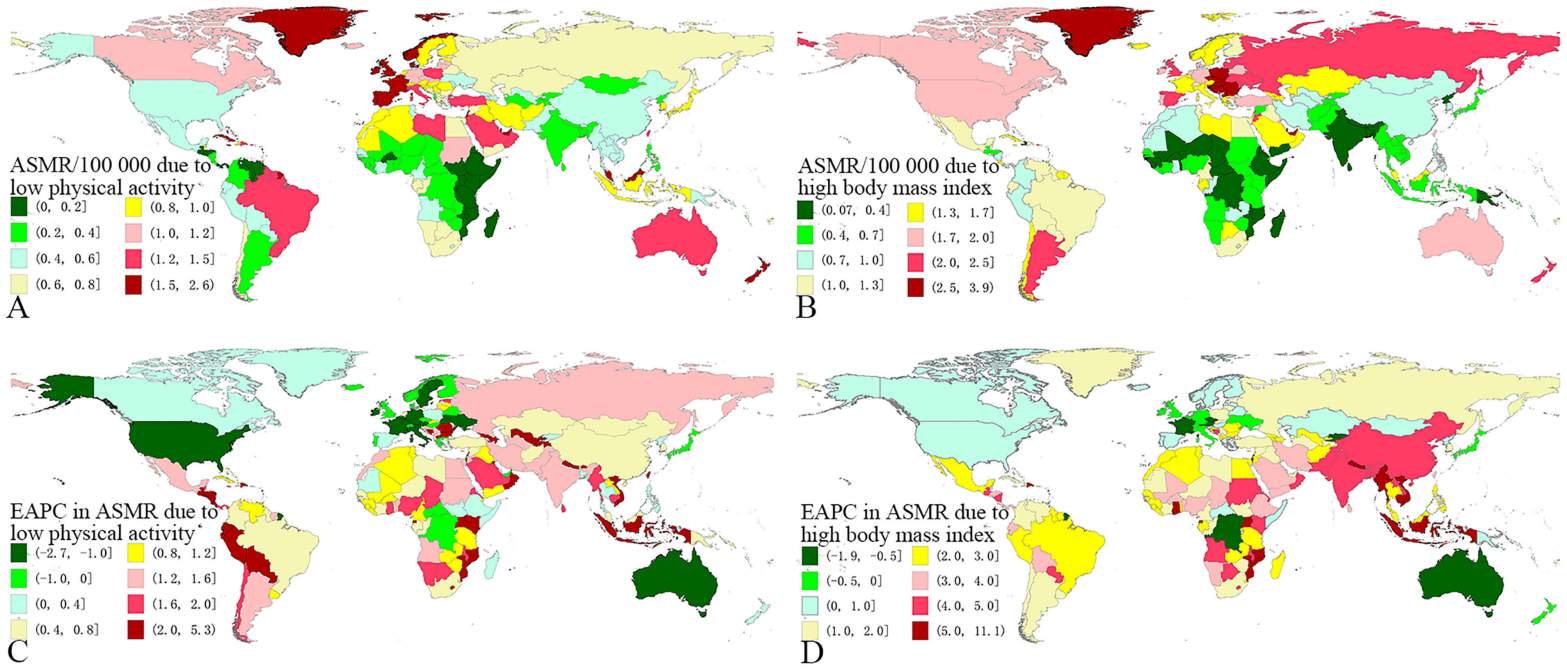
The global distribution of colorectal cancer due to low physical activity and high BMI. **(A)** the ASMR of colorectal cancer due to low physical activity in 2019; **(B)** the ASMR of colorectal cancer due to high BMI in 2019; **(C)** the EAPC of colorectal cancer ASMR due to low physical activity, 1990-2019; **(D)** the EAPC of colorectal cancer ASMR due to high BMI, 1990–2019. ASMR, age-standardized mortality rate; EAPC, estimated annual percentage change; BMI, body mass index.

The variety of CRC ASMR due to LPA was over 40 times across the world in 2019, with the highest rate observed in Barbados (2.55/100,000), and the lowest rate observed in Guatemala (0.06/100,000) (Figure 2A; Supplementary Table 1). The highest ASMR for CRC caused by high BMI was observed in Hungary (3.88/100,000), and the lowest ASMR for CRC caused by high BMI was observed in Somalia (0.07/100,000). The difference between the two was more than 55 times. There were 11 countries and territories with CRC ASMR attributable to high BMI higher than 3/100,000, and 37 countries and territories with CRC ASMR attributable to high BMI higher than 2/100,000 (Figure 2B; Supplementary Table 1).

### Temporal Trends of CRC Deaths Due to LPA and High BMI

In the past three decades, the global number of CRC deaths attributed to LPA increased by 118%, from 26.93 (95% UI 6.79–52.68) thousand to 58.66 (95% UI 16.87–112.15) thousand, whereas the CRC ASMR due to LPA decreased by 6%, from 0.82/100,000 to 0.77/100,000, with an EAPC of−0.39 (95% CI−0.49–−0.29). The CRC death numbers due to high BMI significantly increased by 162%, from 31.90 (95% UI 15.67–53.51) thousand to 85.88 (95% UI 46.85–136.52) thousand, and the CRC ASMR due to high BMI increased by 24%, with an EAPC of 0.64 (95% CI 0.57–0.71) (Figures 1A,B; Table 1). The increasing trend of CRC ASMR due to high BMI was more obvious in males (EAPC = 0.83, 95% CI 0.76–0.90) than in females (EAPC = −0.08, 95% CI−0.17–0.01) and the decreasing trend of CRC ASMR attributable to LPA was more obvious in females (EAPC = −0.64, 95% CI−0.76–−0.52) than in males (EAPC = −0.05, 95% CI−0.13–0.03) (Figure 1B; Table 1). EAPCs had a significant linear relationship with the age increase, and EAPCs decreased below age 70 and increased above age 70. The ASMR caused by high BMI in people under 59 and over 90 showed an upward trend, with EAPCs higher than 0, while the ASMRs caused by LPA showed a downward trend in the 65–79 age group, with EAPCs < 0 (Figure 1D).

The ASMR of CRC due to LPA and high BMI showed an upward trend in most SDI regions, except for high SDI region. The most obvious increasing trend was observed in low-middle SDI region, with EAPC of 1.33 (95% CI 1.27–1.38) for LPA and 3.84 (95% CI 3.79–3.89) for high BMI (Table 1). The ASMRs in most GBD regions (17/21 for LPA and 16/21 for high BMI) had increased significantly. Among them, the region with the fastest increase in ASMR of CRC due to LPA was Andean Latin America (EAPC = 2.43, 95% CI 2.25–2.62), and the region with the fastest increase in ASMR of CRC due to high BMI was East Asia (EAPC = 4.53, 95% CI 4.24–4.81) (Table 1). In the past three decades, the highest EAPC in ASMR of CRC due to LPA was observed in Equatorial Guinea (EAPC = 5.24, 95% CI 4.86–5.63), and the EAPC exceeding 2 was observed in other 33 countries and territories, such as Uzbekistan, Tajikistan, El Salvador, etc. The countries with the fastest decline were Austria (EAPC = −2.65, 95% CI−2.91–−2.39) and the USA (EAPC = −2.46, 95% CI−2.79–−2.13) and the EAPC < −1 was observed only in other 12 countries and territories, such as France, Italy, Germany, Sweden, etc. (Figure 2C; Supplementary Table 1). The highest EAPC in ASMR due to high BMI was also observed in Equatorial Guinea, with an EAPC of 11.03 (95% CI 10.10–11.96), and the EAPC exceeding 5 was observed in other 11 countries and territories, including Vietnam, Nepal, Mozambique, Myanmar, Indonesia, etc. Austria (EAPC = −1.83, 95% CI−1.99 to −1.67) was also the country with the fast decline in ASMR due to high BMI, and the EAPC < −1 was observed only in Austria, DR Congo, Luxembourg, and Israel (Figure 2D; Supplementary Table 1).

### Influential Factors of Temporal Trends of ASMR Attributed to LPA and High BMI

**Figure 3:**
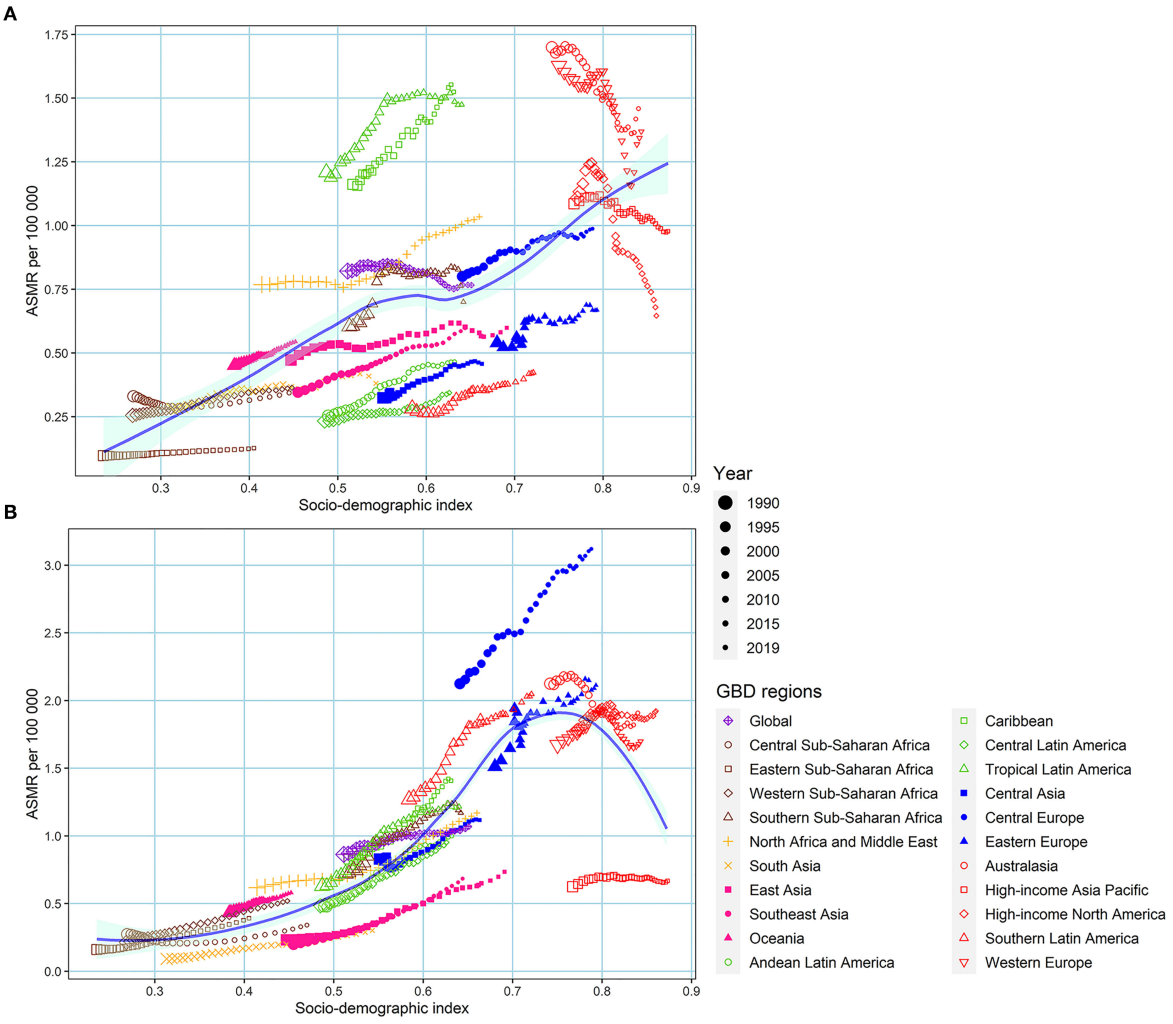
The association between low physical activity and high BMI-induced colorectal cancer ASMR with SDI. **(A)** low physical activity; **(B)** high BMI. ASMR, age-standardized mortality rate; SDI, sociodemographic index; BMI, body mass index.

In most GBD regions (except High-income North America, High-income Asia Pacific, Western Europe, and Australasia), ASMR due to LPA increased or remained relatively stable with the increase of SDI (Figure 3A). ASMR due to high BMI in most GBD regions increased rapidly with the increase of SDI except for a few GBD regions, such as High-income Asia Pacific, and Australasia, which showed a stable situation (Figure 3B). No significant differences were found between males and females (Supplementary Figure 2).

**Figure 4:**
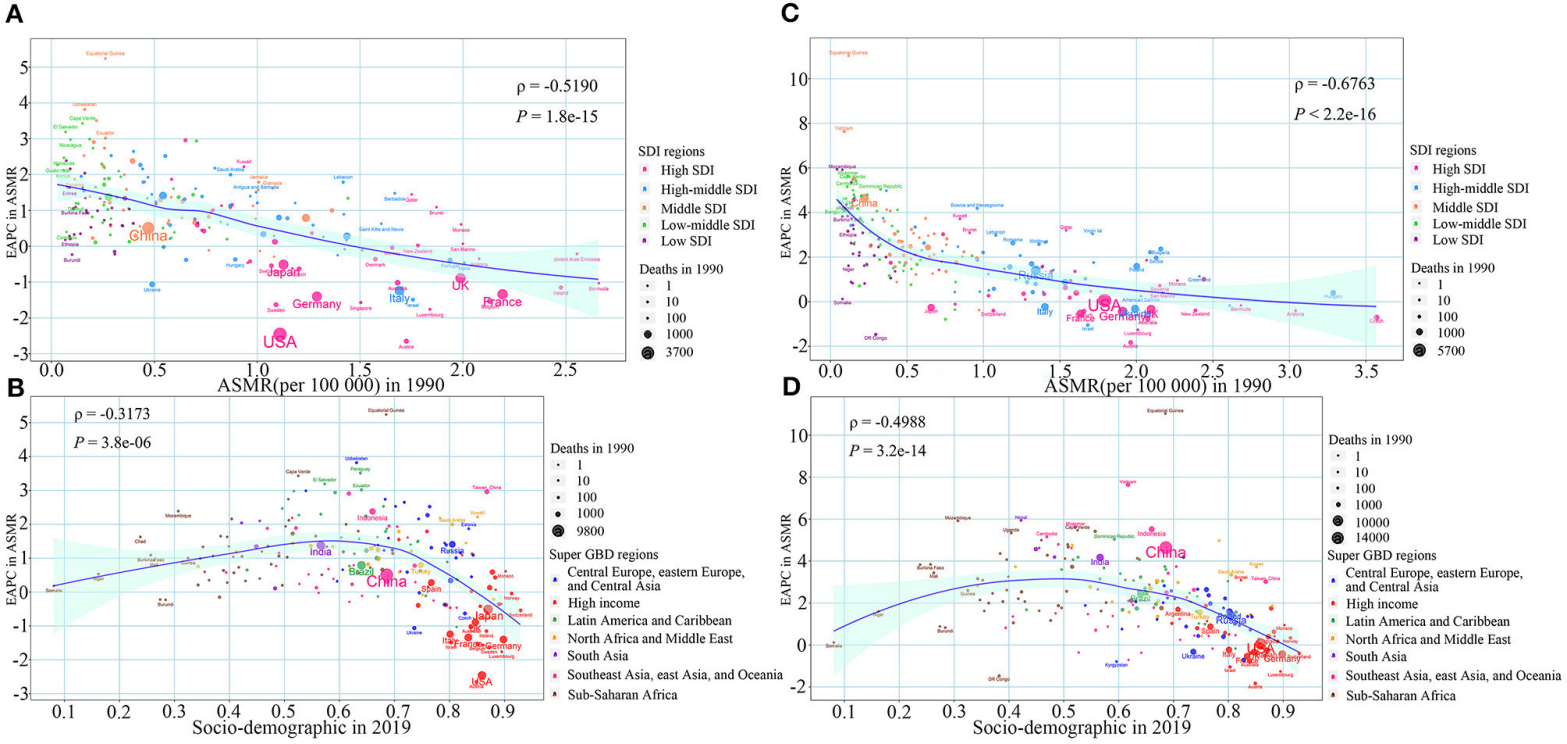
The association between the EAPC of colorectal cancer ASMR and the corresponding ASMR in 1990 and SDI in 2019. **(A)** EAPC of ASMR due to low physical activity and the corresponding ASMR in 1990; **(B)** EAPC of ASMR due to high BMI and the corresponding ASMR in 1990; **(C)** EAPC of ASMR due to low physical activity and the SDI in 2019; **(D)** EAPC of ASMR due to high BMI and the SDI in 2019. The blue line was an adaptive association fitted with adaptive Loess regression based on all data points. EAPC, estimated annual percentage change; ASMR, age-standardized mortality rate; SDI, sociodemographic index; BMI, body mass index.

At the national level, we observed a significant negative correlation between the EAPC of ASMR caused by LPA and the initially ASMR of CRC attributable to LPA in 1990 (ρ = −0.519, *P* = 1.8e-15) (Figure 4A). A negative correlation was also observed between the EAPCs of ASMR caused by LPA and SDI in 2019 (ρ = −0.3173, *P* = 3.8e-06) (Figure 4B). Similarly, significant negative correlations were observed between EAPCs of ASMR caused by high BMI and initial disease burden and SDI in 2019, with ρ of −0.6763 (*P* < 2.2e-16) and−0.4988 (*P* = 3.2e-14), respectively (Figures 4C,D).

## Discussion

Complying with the framework of the GBD 2019 study, we comprehensively assessed the geographic and temporal trends and its potential influencing factors of CRC disease burden caused by LPA and high BMI from 1990 to 2019. In general, the number of CRC deaths caused by high BMI worldwide increased steadily, and the corresponding ASMR also increased during this period, especially in males. Although the ASMR of CRC caused by LPA declined and was more pronounced among females, the corresponding death number was still rising steadily. ASMR caused by LPA in the 65–79 age group showed a downward trend, with EAPCs < 0. In 2019, high-middle and high SDI regions ranked top in ASMRs for CRC due to LPA and high BMI. However, only high SDI region led to a significant decline in ASMR of CRC due to LPA and high BMI. It is worth noting that ASMRs in the middle, low-middle SDI regions have increased significantly. Thirteen countries showed a significant downward trend in CRC ASMR attributed to LPA, with EAPCs < −1. However, only 4 countries showed a significant downward trend in CRC ASMR attributable to high BMI, with EAPCs < −1. Countries with a higher baseline burden in 1990 and a higher SDI in 2019 had a faster decline in ASMR due to high BMI and LPA.

Obesity and LPA are two risk factors associated with CRC (7, 9, 26, 27). Obesity can cause changes in the levels of insulin, IGF-1, leptin, adiponectin, steroid hormones and cytokines in the body, creating a favorable environment for the occurrence and development of tumors (28). Oxidative stress caused by obesity can promote DNA damage and further lead to cancer-related genetic instability (29). In addition, obesity can also promote the occurrence and progression of cancer through inflammation (30). Active physical activity can not only reduce the risk of cancer by reducing weight but also reduce the cancer risk by reducing the level of insulin, IGF-1, IGF-binding protein 3 and leptin in the body. In addition, physical activity may also reduce the incidence of CRC by reducing the contact time between gastrointestinal carcinogens and colon mucosa (27).

With the widespread recommendation of colonoscopy in the late 1990's and changes in CRC-related risk factors, the ASMR of CRC has begun to decline or remain stable in some high-income countries, but the absolute disease burden of CRC is still heavy (31). In 2019, the number of global CRC deaths due to LPA and high BMI were more than twice that in 1990, resulting in 58.66 thousand and 85.88 thousand CRC-related deaths. From 1990 to 2017, the number of people over 30 in the world has almost doubled from 2157.4 million in 1990 to 3825.4 million in 2017 (32). A significantly increasing population will increase the number of CRC caused by high BMI and LPA levels to a certain extent. Economic development and technological changes have changed food prices, economic structure and disposable income, which have led to an inverted U-shaped curve of economic development and obesity (33, 34). As the economic level increases, the lack of physical activity is increasing. A study conducted in 2018 showed that in 2016, the prevalence of insufficient physical activity in high-income areas was about twice that of low-income countries, and it was on the rise (19). The increasing prevalence of obesity and insufficient physical activity also explains to some extent the increase in CRC deaths attributed to LPA and high BMI.

Our study found that males have a higher disease burden of CRC due to LPA and high BMI than females, although females have a higher prevalence of obesity and LPA (35, 36). Compared with males, female' lifestyles may be healthier. Females prefer low-calorie foods, tend to consume large amounts of fruits, vegetables and fiber, smoke less and drink less alcohol (37). Further, the content of estrogen in the body of female is much higher than that of male. There is evidence that the expression of estrogen receptor β (ERβ/ESR2) is inversely related to the presence of colorectal polyps and tumor stage. Phytoestrogens or synthetic ERβ selective agonists can promote cell apoptosis by activating or up-regulating ERβ in the colon (38, 39). This reduces the risk of death from CRC for females. For people at high risk of CRC with LPA and high BMI, weight loss and increased physical activity can be used to reduce the risk of CRC. In addition, the government also needs to adopt health education, increase sports facilities, and advocate increased vegetable intake and physical exercise to reduce the burden of CRC caused by LPA and high BMI.

In our study, high and high-middle SDI regions were found to have the highest ASMRs of CRC due to LPA and high BMI. The ASMRs of CRC attributable to high BMI and LPA showed a downward trend in high SDI region and an upward trend in other SDI regions, especially in middle SDI region. Previous studies found that the incidence and mortality of CRC in many middle-income and low-income countries were still rising rapidly, and highly developed countries tended to stabilize or decline (4, 40, 41). These findings support the views of this research to a certain extent. The high-calorie, low-physical western lifestyle that people in developing countries are adapting to may be an important factor leading to the increase in the incidence and death of CRC in developing countries (42). Although the incidence and death of CRC in high-income countries are declining, the disease burden of CRC in high-income countries is still severe. Countries all over the world need to take some targeted measures, such as health education, advocacy to maintain a healthy weight and active participation in physical exercises, to reduce the burden of CRC caused by LPA and high BMI.

We observed that countries with a higher baseline burden in 1990 had a faster decline in ASMR, and the EAPC of ASMR had a roughly linear relationship with the baseline disease burden. Countries with higher SDI in 2019 also had a faster decline in ASMR, and EAPC has a roughly inverted U-shaped relationship with SDI in 2019. The countries with a heavier baseline disease burden in 1990 were mostly developed countries, such as France, the United Kingdom, and Italy. They could adopt several methods, such as, health education, screening, removal of polyps and early detection efforts, to reduce the disease burden of CRC. Countries with a low baseline disease burden, such as China, are mostly developing countries, and have experienced rapid economic development and increasingly westernized lifestyles in the past 30 years. The increase in obesity and the decrease in physical activity brought about by the westernized lifestyle are some of the important reasons for the increase in CRC caused by LPA and high BMI in these countries (4). The CRC disease burden varies greatly between different countries and territories, and the formulation of CRC prevention and control policies needs to be carried out according to the specific conditions of each region. Developing countries could avoid the increase in the burden of CRC caused by the westernized lifestyle by adopting measures that can help local residents establish a healthy lifestyle, such as actively promoting healthy diets and the health benefits of active participation in sports activities. For developed countries, under the premise of adhering to previous screening, early diagnosis and other measures, more targeted measures need to be taken to reduce the prevalence of obesity and low physical activity in the local area.

Our study has some strengths. First, the data used in our study is calculated using a robust method using currently all available data, and its quality is currently the best. Second, the association between risk factors and CRC is reliable because the association was obtained by analyzing data from prospective observational studies using meta-analysis. Third, our study is the latest detailed description of the geographical and temporal trends and its influencing factors in the global burden of CRC caused by high BMI and LPA, which can provide a basis for the prevention and control of CRC. However, our research has some limitations. First, because the GBD estimation of CRC is based on a large number of different quality data using mathematical models, there may be a certain deviation between the estimated value and the actual data. Second, although GBD researchers use different data including cancer registration, vital registration, and oral autopsy to estimate the burden of cancer disease, some countries still do not have any of these sources. Using predictive covariates or trends from neighboring countries to predict the burden of disease in these countries will cause unavoidable bias. In addition, there are differences in the quality and quantity of data sources, the evaluation of the cause, and the diagnostic accuracy of CRC in different regions, which may cause heterogeneity (14, 43). Third, some changes in ASMRs may be caused by detection deviations and changes in screening schemes. Finally, for some countries with small populations, small changes in the number of CRC cases may cause huge changes in ASMR, and there may be potential biases in the estimates of these countries.

## Conclusions

In summary, the global burden of CRC due to LPA and high BMI has been on the rise in the past 30 years and is likely to rise in the future. The rising trend of the corresponding ASMRs in low-middle, middle SDI regions is particularly obvious. Countries in the world, especially those in the low-middle, middle, and high-middle SDI regions, should take active measures to deal with the rising burden of CRC caused by LPA and high BMI.

## Data Availability Statement

The datasets presented in this study can be extracted from the online database (http://ghdx.healthdata.org/gbd-results-tool).

## Ethics Statement

The studies involving human participants were reviewed and approved by the Institutional Review Boards of Qilu Hospital of Shandong University with approval number KYLL-202011(KS)-239. Written informed consent for participation was not required for this study in accordance with the national legislation and the institutional requirements.

## Author Contributions

JM, XYa, and ML: conceptualization. JM, TZ, XYi, YZ, XZ, and JC: data curation, methodology, and formal analysis. JM, TZ, XYi, HC, YZ, and XYa: formal analysis, software, and visualization. JM: writing original draft. XYa and ML: funding acquisition and writing review and editing. All authors approved the final manuscript for submission.

## Funding

This work was supported by the National Natural Science Foundation of China (Grant Numbers: 82103912, 82173591, and 81973116), the China Postdoctoral Science Foundation (2021M700080), the Shandong Provincial Natural Science Foundation (Grant Number: ZR2020QH302), and the National Key Research and Development Program of China (Grant Number: 2017YFC0907003). The funders were not involved in the collection, analysis, or interpretation of data, or the writing or submitting of this report.

## Conflict of Interest

The authors declare that the research was conducted in the absence of any commercial or financial relationships that could be construed as a potential conflict of interest.

## Publisher's Note

All claims expressed in this article are solely those of the authors and do not necessarily represent those of their affiliated organizations, or those of the publisher, the editors and the reviewers. Any product that may be evaluated in this article, or claim that may be made by its manufacturer, is not guaranteed or endorsed by the publisher.

## Abbreviations

CRC: colorectal cancer
LPA: low physical activity
BMI: body mass index
ASMR: age-standardized mortality rate
CI: confidence interval
EAPC: estimated annual percentage change
GBD: The Global Burden of Diseases
SDI: sociodemographic index
UI: uncertainty interval
PAF: population attributable fraction
DALY: disability-adjusted life year.
