# Supplementary material for: Spatiotemporal Trends of Colorectal Cancer Mortality Due to Low Physical Activity and High Body Mass Index From 1990 to 2019: A Global, Regional and National Analysis
Source: Front Med (Lausanne). 2022 Jan 10;8:800426. doi: 10.3389/fmed.2021.800426 (PMC8784601; doi:10.3389/fmed.2021.800426)
Supplement: Supplementary file 1 [file Data_Sheet_1.docx]

Supplementary Material

# Supplementary Figures and Tables

## Supplementary Tables

**Supplementary Table1. Death cases and ASMR of colorectal cancer due to low physical activity and high BMI in 1990 and 2019 and the temporal trends from 1990 to 2019 in 204 countries.**

| Characteristics | 1990 | | | | |  | 2019 | | | | |  | EAPC (1990–2019) | | |
| --- | --- | --- | --- | --- | --- | --- | --- | --- | --- | --- | --- | --- | --- | --- | --- |
|  | High BMI | |  | Low activity | |  | High BMI | |  | Low activity | |  | High BMI |  | Low activity |
|  | Death cases | ASMR |  | Death cases | ASMR |  | Death cases | ASMR |  | Death cases | ASMR |  |  |  |  |
|  | Estimate×10^3^  (95% UI) | Estimate×10^-5^  (95% UI) |  | Estimate×10^3^  (95% UI) | Estimate×10^-5^  (95% UI) |  | Estimate×10^3^  (95% UI) | Estimate×10^-5^  (95% UI) |  | Estimate×10^3^  (95% UI) | Estimate×10^-5^  (95% UI) |  | Estimate  (95% CI) |  | Estimate  (95% CI) |
| Afghanistan | 0.02  (0.01, 0.05) | 0.31  (0.10, 0.69) |  | 0.04  (0.01, 0.09) | 0.70  (0.22, 1.34) |  | 0.07  (0.03, 0.12) | 0.55  (0.28, 0.99) |  | 0.08  (0.02, 0.16) | 0.80  (0.26, 1.52) |  | 2.81  (1.88, 3.74) |  | 0.76  (0.47, 1.05) |
| Albania | 0.01  (0.01, 0.02) | 0.65  (0.31, 1.07) |  | 0.00  (0.00, 0.01) | 0.26  (0.08, 0.52) |  | 0.04  (0.02, 0.07) | 0.96  (0.48, 1.63) |  | 0.01  (0.00, 0.03) | 0.33  (0.10, 0.67) |  | 2.02  (1.62, 2.43) |  | 1.23  (0.98, 1.48) |
| Algeria | 0.05  (0.02, 0.09) | 0.47  (0.22, 0.82) |  | 0.07  (0.02, 0.12) | 0.76  (0.29, 1.30) |  | 0.27  (0.14, 0.44) | 0.90  (0.47, 1.43) |  | 0.26  (0.10, 0.44) | 0.93  (0.36, 1.57) |  | 2.45  (2.35, 2.55) |  | 0.82  (0.76, 0.87) |
| American Samoa | 0.00  (0.00, 0.00) | 2.03  (1.16, 3.08) |  | 0.00  (0.00, 0.00) | 1.32  (0.42, 2.36) |  | 0.00  (0.00, 0.00) | 2.23  (1.31, 3.31) |  | 0.00  (0.00, 0.00) | 1.44  (0.45, 2.61) |  | 0.25  (0.17, 0.33) |  | 0.26  (0.19, 0.33) |
| Andorra | 0.00  (0.00, 0.00) | 3.04  (1.38, 5.37) |  | 0.00  (0.00, 0.00) | 2.08  (0.44, 4.08) |  | 0.00  (0.00, 0.01) | 2.69  (1.35, 4.49) |  | 0.00  (0.00, 0.01) | 1.90  (0.46, 3.58) |  | -0.40  (-0.50, -0.31) |  | -0.35  (-0.39, -0.31) |
| Angola | 0.01  (0.00, 0.01) | 0.14  (0.02, 0.37) |  | 0.01  (0.00, 0.02) | 0.32  (0.09, 0.66) |  | 0.05  (0.02, 0.08) | 0.41  (0.17, 0.77) |  | 0.04  (0.01, 0.07) | 0.46  (0.13, 0.92) |  | 4.28  (3.86, 4.69) |  | 1.36  (1.20, 1.52) |
| Antigua and Barbuda | 0.00  (0.00, 0.00) | 0.60  (0.25, 1.08) |  | 0.00  (0.00, 0.00) | 0.99  (0.27, 1.83) |  | 0.00  (0.00, 0.00) | 1.16  (0.59, 1.93) |  | 0.00  (0.00, 0.00) | 1.59  (0.49, 2.89) |  | 2.31  (2.09, 2.52) |  | 1.51  (1.35, 1.67) |
| Argentina | 0.42  (0.18, 0.73) | 1.31  (0.57, 2.31) |  | 0.05  (0.02, 0.14) | 0.16  (0.06, 0.45) |  | 1.21  (0.63, 1.90) | 2.23  (1.16, 3.50) |  | 0.12  (0.04, 0.29) | 0.21  (0.07, 0.53) |  | 1.70  (1.44, 1.97) |  | 1.55  (1.16, 1.94) |
| Armenia | 0.02  (0.01, 0.04) | 0.87  (0.43, 1.47) |  | 0.01  (0.00, 0.02) | 0.36  (0.11, 0.77) |  | 0.06  (0.03, 0.09) | 1.49  (0.84, 2.27) |  | 0.02  (0.01, 0.04) | 0.54  (0.16, 1.08) |  | 2.24  (1.95, 2.53) |  | 1.59  (1.42, 1.77) |
| Australia | 0.40  (0.21, 0.63) | 2.07  (1.09, 3.26) |  | 0.32  (0.07, 0.63) | 1.68  (0.39, 3.24) |  | 0.78  (0.44, 1.16) | 1.84  (1.06, 2.72) |  | 0.64  (0.17, 1.18) | 1.41  (0.36, 2.61) |  | -0.78  (-0.93, -0.64) |  | -1.02  (-1.15, -0.88) |
| Austria | 0.23  (0.11, 0.38) | 1.96  (0.96, 3.21) |  | 0.22  (0.06, 0.42) | 1.72  (0.46, 3.33) |  | 0.25  (0.12, 0.40) | 1.33  (0.68, 2.11) |  | 0.20  (0.06, 0.38) | 0.96  (0.26, 1.85) |  | -1.83  (-1.99, -1.67) |  | -2.65  (-2.91, -2.39) |
| Azerbaijan | 0.04  (0.02, 0.07) | 0.76  (0.37, 1.26) |  | 0.01  (0.00, 0.02) | 0.26  (0.07, 0.54) |  | 0.12  (0.07, 0.20) | 1.32  (0.69, 2.08) |  | 0.03  (0.01, 0.06) | 0.44  (0.13, 0.85) |  | 2.82  (2.00, 3.66) |  | 2.74  (2.17, 3.31) |
| Bahamas | 0.00  (0.00, 0.00) | 1.46  (0.76, 2.34) |  | 0.00  (0.00, 0.00) | 1.42  (0.39, 2.62) |  | 0.01  (0.00, 0.01) | 1.93  (1.01, 3.11) |  | 0.01  (0.00, 0.01) | 1.69  (0.51, 2.99) |  | 1.42  (1.26, 1.57) |  | 0.89  (0.73, 1.04) |
| Bahrain | 0.00  (0.00, 0.00) | 1.39  (0.74, 2.18) |  | 0.00  (0.00, 0.00) | 1.42  (0.56, 2.39) |  | 0.01  (0.01, 0.02) | 1.66  (0.90, 2.56) |  | 0.01  (0.00, 0.02) | 1.43  (0.57, 2.4) |  | 0.43  (0.14, 0.73) |  | -0.08  (-0.39, 0.24) |
| Bangladesh | 0.02  (0.00, 0.06) | 0.05  (0.01, 0.13) |  | 0.08  (0.02, 0.17) | 0.21  (0.05, 0.46) |  | 0.18  (0.07, 0.38) | 0.14  (0.05, 0.30) |  | 0.28  (0.07, 0.61) | 0.25  (0.07, 0.54) |  | 4.19  (4.00, 4.39) |  | 0.11  (-0.18, 0.41) |
| Barbados | 0.00  (0.00, 0.01) | 1.39  (0.67, 2.25) |  | 0.01  (0.00, 0.01) | 1.67  (0.54, 2.94) |  | 0.01  (0.01, 0.02) | 2.31  (1.22, 3.70) |  | 0.01  (0.00, 0.02) | 2.55  (0.88, 4.48) |  | 1.60  (1.38, 1.82) |  | 1.48  (1.22, 1.74) |
| Belarus | 0.17  (0.09, 0.28) | 1.32  (0.68, 2.13) |  | 0.07  (0.02, 0.14) | 0.55  (0.17, 1.10) |  | 0.29  (0.16, 0.47) | 1.84  (0.97, 2.92) |  | 0.11  (0.03, 0.21) | 0.66  (0.20, 1.30) |  | 0.43  (0.13, 0.72) |  | -0.02  (-0.33, 0.30) |
| Belgium | 0.26  (0.13, 0.43) | 1.66  (0.83, 2.77) |  | 0.34  (0.09, 0.65) | 2.13  (0.57, 4.03) |  | 0.37  (0.19, 0.60) | 1.53  (0.79, 2.46) |  | 0.42  (0.12, 0.74) | 1.53  (0.43, 2.77) |  | -0.43  (-0.50, -0.35) |  | -1.53  (-1.78, -1.29) |
| Belize | 0.00  (0.00, 0.00) | 0.40  (0.15, 0.73) |  | 0.00  (0.00, 0.00) | 0.59  (0.17, 1.09) |  | 0.00  (0.00, 0.00) | 1.16  (0.63, 1.77) |  | 0.00  (0.00, 0.00) | 0.95  (0.28, 1.69) |  | 3.40  (2.66, 4.14) |  | 1.61  (1.06, 2.16) |
| Benin | 0.00  (0.00, 0.01) | 0.21  (0.07, 0.42) |  | 0.00  (0.00, 0.01) | 0.24  (0.06, 0.49) |  | 0.02  (0.01, 0.04) | 0.49  (0.24, 0.86) |  | 0.01  (0.00, 0.03) | 0.33  (0.09, 0.67) |  | 3.09  (2.88, 3.29) |  | 1.33  (1.22, 1.44) |
| Bermuda | 0.00  (0.00, 0.00) | 2.68  (1.38, 4.13) |  | 0.00  (0.00, 0.00) | 2.66  (0.83, 4.69) |  | 0.00  (0.00, 0.00) | 2.36  (1.30, 3.67) |  | 0.00  (0.00, 0.00) | 1.98  (0.67, 3.49) |  | -0.17  (-0.40, 0.05) |  | -1.03  (-1.12, -0.95) |
| Bhutan | 0.00  (0.00, 0.00) | 0.10  (0.02, 0.26) |  | 0.00  (0.00, 0.00) | 0.33  (0.10, 0.67) |  | 0.00  (0.00, 0.00) | 0.35  (0.14, 0.69) |  | 0.00  (0.00, 0.01) | 0.56  (0.18, 1.09) |  | 4.62  (4.47, 4.77) |  | 2.10  (1.99, 2.21) |
| Bolivia | 0.01  (0.01, 0.03) | 0.44  (0.17, 0.82) |  | 0.01  (0.00, 0.01) | 0.24  (0.05, 0.55) |  | 0.09  (0.05, 0.16) | 1.09  (0.52, 1.88) |  | 0.04  (0.01, 0.08) | 0.49  (0.10, 1.03) |  | 3.15  (3.06, 3.24) |  | 2.30  (2.24, 2.36) |
| Bosnia and Herzegovina | 0.04  (0.02, 0.06) | 0.96  (0.46, 1.62) |  | 0.01  (0.00, 0.03) | 0.40  (0.11, 0.85) |  | 0.15  (0.08, 0.25) | 2.54  (1.36, 4.14) |  | 0.05  (0.01, 0.10) | 0.79  (0.21, 1.61) |  | 4.19  (3.69, 4.70) |  | 2.65  (2.15, 3.15) |
| Botswana | 0.00  (0.00, 0.00) | 0.37  (0.13, 0.74) |  | 0.00  (0.00, 0.00) | 0.45  (0.12, 0.90) |  | 0.02  (0.01, 0.03) | 1.31  (0.69, 2.15) |  | 0.01  (0.00, 0.02) | 0.80  (0.21, 1.55) |  | 4.13  (3.77, 4.49) |  | 1.80  (1.52, 2.08) |
| Brazil | 0.54  (0.26, 0.93) | 0.64  (0.30, 1.11) |  | 0.96  (0.31, 1.63) | 1.24  (0.44, 2.06) |  | 2.86  (1.64, 4.30) | 1.22  (0.70, 1.84) |  | 3.42  (1.42, 5.54) | 1.50  (0.63, 2.41) |  | 2.43  (2.15, 2.71) |  | 0.79  (0.62, 0.97) |
| Brunei | 0.00  (0.00, 0.00) | 0.91  (0.23, 2.07) |  | 0.00  (0.00, 0.00) | 1.87  (0.38, 3.77) |  | 0.00  (0.00, 0.01) | 1.63  (0.71, 2.90) |  | 0.00  (0.00, 0.01) | 2.08  (0.44, 4.12) |  | 3.09  (2.67, 3.52) |  | 1.09  (0.81, 1.37) |
| Bulgaria | 0.27  (0.15, 0.41) | 2.16  (1.19, 3.25) |  | 0.06  (0.02, 0.13) | 0.55  (0.16, 1.14) |  | 0.44  (0.23, 0.70) | 3.04  (1.62, 4.87) |  | 0.12  (0.04, 0.25) | 0.79  (0.24, 1.64) |  | 2.37  (1.86, 2.89) |  | 2.52  (1.89, 3.15) |
| Burkina Faso | 0.00  (0.00, 0.01) | 0.11  (0.03, 0.28) |  | 0.00  (0.00, 0.01) | 0.12  (0.03, 0.26) |  | 0.03  (0.01, 0.05) | 0.32  (0.13, 0.62) |  | 0.01  (0.00, 0.03) | 0.17  (0.05, 0.37) |  | 3.84  (3.76, 3.92) |  | 1.09  (0.97, 1.21) |
| Burundi | 0.00  (0.00, 0.01) | 0.14  (0.04, 0.34) |  | 0.00  (0.00, 0.01) | 0.10  (0.03, 0.27) |  | 0.01  (0.00, 0.02) | 0.19  (0.06, 0.43) |  | 0.00  (0.00, 0.01) | 0.10  (0.03, 0.26) |  | 0.83  (0.65, 1.01) |  | -0.23  (-0.28, -0.17) |
| Cambodia | 0.00  (0.00, 0.01) | 0.11  (0.02, 0.30) |  | 0.01  (0.00, 0.02) | 0.26  (0.06, 0.58) |  | 0.05  (0.02, 0.10) | 0.41  (0.16, 0.81) |  | 0.04  (0.01, 0.08) | 0.43  (0.10, 0.92) |  | 5.16  (4.88, 5.44) |  | 1.88  (1.79, 1.98) |
| Cameroon | 0.02  (0.01, 0.04) | 0.59  (0.25, 1.06) |  | 0.01  (0.00, 0.03) | 0.38  (0.10, 0.75) |  | 0.11  (0.06, 0.19) | 1.02  (0.52, 1.72) |  | 0.04  (0.01, 0.09) | 0.50  (0.13, 1.01) |  | 1.88  (1.79, 1.97) |  | 1.06  (1.00, 1.11) |
| Canada | 0.52  (0.27, 0.82) | 1.61  (0.83, 2.54) |  | 0.35  (0.08, 0.72) | 1.09  (0.24, 2.21) |  | 1.28  (0.69, 1.94) | 1.82  (1.00, 2.75) |  | 0.82  (0.20, 1.60) | 1.08  (0.26, 2.12) |  | 0.40  (0.25, 0.54) |  | 0.13  (0.02, 0.23) |
| Cape Verde | 0.00  (0.00, 0.00) | 0.14  (0.05, 0.28) |  | 0.00  (0.00, 0.00) | 0.15  (0.04, 0.31) |  | 0.00  (0.00, 0.01) | 0.82  (0.41, 1.36) |  | 0.00  (0.00, 0.00) | 0.48  (0.14, 0.96) |  | 5.47  (5.05, 5.89) |  | 3.43  (3.01, 3.85) |
| Central African Republic | 0.00  (0.00, 0.00) | 0.13  (0.03, 0.33) |  | 0.00  (0.00, 0.01) | 0.31  (0.08, 0.64) |  | 0.00  (0.00, 0.01) | 0.16  (0.05, 0.35) |  | 0.00  (0.00, 0.01) | 0.28  (0.07, 0.59) |  | 0.88  (0.73, 1.03) |  | -0.22  (-0.28, -0.16) |
| Chad | 0.00  (0.00, 0.01) | 0.08  (0.02, 0.19) |  | 0.00  (0.00, 0.01) | 0.15  (0.04, 0.33) |  | 0.01  (0.00, 0.02) | 0.23  (0.09, 0.45) |  | 0.01  (0.00, 0.02) | 0.22  (0.06, 0.48) |  | 3.83  (3.73, 3.92) |  | 1.63  (1.52, 1.73) |
| Chile | 0.09  (0.04, 0.15) | 0.96  (0.46, 1.59) |  | 0.04  (0.01, 0.08) | 0.45  (0.09, 0.95) |  | 0.38  (0.20, 0.58) | 1.57  (0.83, 2.43) |  | 0.17  (0.04, 0.34) | 0.71  (0.15, 1.41) |  | 1.87  (1.76, 1.98) |  | 1.79  (1.63, 1.96) |
| China | 1.82  (0.40, 4.35) | 0.22  (0.05, 0.53) |  | 2.88  (0.74, 5.86) | 0.47  (0.13, 0.95) |  | 14.15  (5.48, 26.65) | 0.72  (0.28, 1.36) |  | 9.83  (2.60, 19.96) | 0.58  (0.16, 1.16) |  | 4.64  (4.33, 4.94) |  | 0.51  (0.36, 0.67) |
| Colombia | 0.08  (0.04, 0.14) | 0.49  (0.22, 0.86) |  | 0.04  (0.01, 0.09) | 0.29  (0.06, 0.65) |  | 0.44  (0.23, 0.75) | 0.83  (0.42, 1.40) |  | 0.19  (0.04, 0.41) | 0.36  (0.07, 0.76) |  | 1.74  (1.52, 1.95) |  | 0.57  (0.48, 0.66) |
| Comoros | 0.00  (0.00, 0.00) | 0.24  (0.07, 0.52) |  | 0.00  (0.00, 0.00) | 0.07  (0.03, 0.23) |  | 0.00  (0.00, 0.00) | 0.40  (0.17, 0.75) |  | 0.00  (0.00, 0.00) | 0.09  (0.03, 0.27) |  | 1.63  (1.47, 1.80) |  | 0.40  (0.28, 0.53) |
| Congo | 0.01  (0.00, 0.01) | 0.48  (0.16, 0.97) |  | 0.00  (0.00, 0.01) | 0.54  (0.14, 1.09) |  | 0.02  (0.01, 0.03) | 0.79  (0.39, 1.35) |  | 0.01  (0.00, 0.02) | 0.62  (0.16, 1.19) |  | 1.62  (1.37, 1.87) |  | 0.51  (0.31, 0.71) |
| Cook Islands | 0.00  (0.00, 0.00) | 0.81  (0.43, 1.29) |  | 0.00  (0.00, 0.00) | 0.58  (0.17, 1.05) |  | 0.00  (0.00, 0.00) | 1.00  (0.56, 1.50) |  | 0.00  (0.00, 0.00) | 0.65  (0.20, 1.20) |  | 0.43  (0.29, 0.58) |  | 0.21  (0.08, 0.35) |
| Costa Rica | 0.01  (0.01, 0.02) | 0.68  (0.32, 1.13) |  | 0.00  (0.00, 0.01) | 0.16  (0.04, 0.37) |  | 0.08  (0.04, 0.14) | 1.57  (0.79, 2.64) |  | 0.02  (0.00, 0.04) | 0.33  (0.07, 0.76) |  | 3.05  (2.90, 3.20) |  | 2.53  (2.36, 2.69) |
| Cote d'Ivoire | 0.02  (0.01, 0.03) | 0.39  (0.15, 0.76) |  | 0.01  (0.00, 0.02) | 0.35  (0.08, 0.73) |  | 0.06  (0.03, 0.10) | 0.60  (0.28, 1.03) |  | 0.03  (0.01, 0.06) | 0.41  (0.11, 0.82) |  | 1.06  (0.91, 1.21) |  | 0.57  (0.45, 0.69) |
| Croatia | 0.13  (0.07, 0.20) | 2.09  (1.11, 3.26) |  | 0.04  (0.01, 0.09) | 0.73  (0.22, 1.46) |  | 0.31  (0.17, 0.48) | 3.39  (1.86, 5.34) |  | 0.09  (0.03, 0.18) | 0.92  (0.27, 1.89) |  | 2.22  (1.97, 2.47) |  | 1.39  (1.13, 1.64) |
| Cuba | 0.10  (0.05, 0.16) | 0.96  (0.50, 1.55) |  | 0.14  (0.04, 0.25) | 1.36  (0.40, 2.46) |  | 0.30  (0.16, 0.48) | 1.57  (0.83, 2.51) |  | 0.37  (0.12, 0.64) | 1.83  (0.60, 3.19) |  | 1.95  (1.67, 2.23) |  | 1.00  (0.87, 1.12) |
| Cyprus | 0.01  (0.00, 0.01) | 0.71  (0.31, 1.26) |  | 0.01  (0.00, 0.01) | 0.83  (0.18, 1.65) |  | 0.02  (0.01, 0.03) | 1.04  (0.52, 1.72) |  | 0.02  (0.00, 0.04) | 1.03  (0.23, 2.03) |  | 1.43  (1.12, 1.74) |  | 1.13  (0.86, 1.40) |
| Czech | 0.49  (0.27, 0.75) | 3.57  (1.96, 5.40) |  | 0.17  (0.05, 0.32) | 1.20  (0.35, 2.35) |  | 0.66  (0.38, 1.02) | 3.08  (1.77, 4.73) |  | 0.23  (0.07, 0.45) | 1.02  (0.30, 2.03) |  | -0.71  (-0.96, -0.47) |  | -0.63  (-0.90, -0.37) |
| Denmark | 0.13  (0.06, 0.21) | 1.56  (0.77, 2.60) |  | 0.14  (0.03, 0.27) | 1.58  (0.36, 3.07) |  | 0.22  (0.11, 0.35) | 1.84  (0.95, 2.95) |  | 0.21  (0.05, 0.39) | 1.61  (0.39, 3.11) |  | 0.18  (-0.09, 0.45) |  | -0.36  (-0.69, -0.03) |
| Djibouti | 0.00  (0.00, 0.00) | 0.15  (0.03, 0.38) |  | 0.00  (0.00, 0.00) | 0.12  (0.04, 0.3) |  | 0.00  (0.00, 0.01) | 0.52  (0.22, 0.98) |  | 0.00  (0.00, 0.00) | 0.17  (0.05, 0.41) |  | 5.00  (4.75, 5.24) |  | 1.20  (1.12, 1.28) |
| Dominica | 0.00  (0.00, 0.00) | 0.83  (0.42, 1.39) |  | 0.00  (0.00, 0.00) | 0.97  (0.26, 1.76) |  | 0.00  (0.00, 0.00) | 1.60  (0.86, 2.59) |  | 0.00  (0.00, 0.00) | 1.36  (0.39, 2.51) |  | 2.47  (2.33, 2.62) |  | 1.40  (1.29, 1.51) |
| Dominican Republic | 0.01  (0.00, 0.02) | 0.33  (0.12, 0.64) |  | 0.02  (0.01, 0.04) | 0.71  (0.23, 1.25) |  | 0.09  (0.04, 0.16) | 1.01  (0.46, 1.78) |  | 0.11  (0.04, 0.20) | 1.30  (0.44, 2.31) |  | 5.04  (4.67, 5.42) |  | 2.94  (2.69, 3.19) |
| DR Congo | 0.04  (0.02, 0.08) | 0.30  (0.13, 0.56) |  | 0.03  (0.01, 0.07) | 0.31  (0.08, 0.65) |  | 0.08  (0.03, 0.17) | 0.25  (0.10, 0.50) |  | 0.08  (0.02, 0.19) | 0.31  (0.07, 0.70) |  | -1.47  (-2.32, -0.61) |  | -0.15  (-0.52, 0.21) |
| Ecuador | 0.03  (0.01, 0.04) | 0.53  (0.27, 0.87) |  | 0.01  (0.00, 0.02) | 0.26  (0.05, 0.55) |  | 0.17  (0.09, 0.27) | 1.18  (0.62, 1.87) |  | 0.07  (0.02, 0.14) | 0.54  (0.12, 1.07) |  | 3.24  (2.95, 3.53) |  | 3.02  (2.72, 3.32) |
| Egypt | 0.13  (0.07, 0.21) | 0.45  (0.22, 0.72) |  | 0.13  (0.04, 0.23) | 0.53  (0.18, 0.91) |  | 0.65  (0.35, 1.10) | 1.01  (0.54, 1.69) |  | 0.43  (0.14, 0.79) | 0.78  (0.27, 1.41) |  | 2.70  (2.58, 2.82) |  | 1.29  (1.19, 1.40) |
| El Salvador | 0.01  (0.00, 0.01) | 0.27  (0.12, 0.48) |  | 0.00  (0.00, 0.00) | 0.07  (0.02, 0.16) |  | 0.05  (0.02, 0.08) | 0.83  (0.41, 1.42) |  | 0.01  (0.00, 0.03) | 0.17  (0.04, 0.39) |  | 3.68  (3.09, 4.28) |  | 3.19  (2.79, 3.60) |
| Equatorial Guinea | 0.00  (0.00, 0.00) | 0.12  (0.02, 0.31) |  | 0.00  (0.00, 0.00) | 0.26  (0.07, 0.54) |  | 0.01  (0.00, 0.01) | 1.37  (0.68, 2.37) |  | 0.00  (0.00, 0.01) | 0.88  (0.24, 1.65) |  | 11.03  (10.10, 11.96) |  | 5.24  (4.86, 5.63) |
| Eritrea | 0.00  (0.00, 0.00) | 0.09  (0.03, 0.20) |  | 0.00  (0.00, 0.00) | 0.09  (0.03, 0.22) |  | 0.01  (0.00, 0.01) | 0.32  (0.14, 0.58) |  | 0.00  (0.00, 0.01) | 0.15  (0.04, 0.35) |  | 4.07  (3.47, 4.67) |  | 1.63  (1.34, 1.91) |
| Estonia | 0.03  (0.02, 0.05) | 1.60  (0.88, 2.50) |  | 0.01  (0.00, 0.02) | 0.58  (0.18, 1.20) |  | 0.06  (0.04, 0.10) | 2.22  (1.27, 3.49) |  | 0.03  (0.01, 0.05) | 0.82  (0.25, 1.59) |  | 1.36  (1.09, 1.64) |  | 1.87  (1.54, 2.19) |
| Eswatini | 0.00  (0.00, 0.00) | 0.86  (0.43, 1.45) |  | 0.00  (0.00, 0.00) | 0.47  (0.11, 0.93) |  | 0.01  (0.00, 0.01) | 1.58  (0.80, 2.54) |  | 0.00  (0.00, 0.01) | 0.73  (0.19, 1.46) |  | 2.02  (1.46, 2.59) |  | 1.34  (0.87, 1.81) |
| Ethiopia | 0.02  (0.00, 0.07) | 0.11  (0.02, 0.33) |  | 0.01  (0.00, 0.04) | 0.09  (0.02, 0.26) |  | 0.11  (0.05, 0.23) | 0.27  (0.11, 0.58) |  | 0.03  (0.01, 0.10) | 0.10  (0.03, 0.28) |  | 3.16  (2.59, 3.74) |  | 0.21  (0.07, 0.35) |
| Fiji | 0.00  (0.00, 0.00) | 0.73  (0.32, 1.24) |  | 0.00  (0.00, 0.00) | 0.58  (0.16, 1.11) |  | 0.01  (0.00, 0.01) | 1.25  (0.66, 1.96) |  | 0.00  (0.00, 0.01) | 0.85  (0.25, 1.61) |  | 1.78  (1.71, 1.86) |  | 1.37  (1.30, 1.44) |
| Finland | 0.07  (0.04, 0.12) | 1.03  (0.51, 1.71) |  | 0.07  (0.02, 0.14) | 0.97  (0.22, 1.91) |  | 0.14  (0.07, 0.23) | 1.14  (0.59, 1.81) |  | 0.13  (0.03, 0.24) | 0.91  (0.22, 1.73) |  | 0.27  (0.24, 0.30) |  | -0.42  (-0.62, -0.23) |
| France | 1.38  (0.69, 2.28) | 1.63  (0.82, 2.69) |  | 1.96  (0.56, 3.54) | 2.19  (0.61, 3.98) |  | 2.18  (1.14, 3.51) | 1.48  (0.78, 2.35) |  | 2.74  (0.91, 4.81) | 1.64  (0.50, 2.91) |  | -0.53  (-0.61, -0.44) |  | -1.34  (-1.50, -1.18) |
| Gabon | 0.00  (0.00, 0.01) | 0.58  (0.19, 1.32) |  | 0.00  (0.00, 0.01) | 0.57  (0.16, 1.21) |  | 0.01  (0.01, 0.02) | 1.40  (0.74, 2.30) |  | 0.01  (0.00, 0.01) | 0.74  (0.21, 1.40) |  | 2.87  (2.62, 3.13) |  | 0.72  (0.62, 0.83) |
| Gambia | 0.00  (0.00, 0.00) | 0.15  (0.05, 0.31) |  | 0.00  (0.00, 0.00) | 0.16  (0.04, 0.34) |  | 0.00  (0.00, 0.01) | 0.40  (0.18, 0.70) |  | 0.00  (0.00, 0.00) | 0.25  (0.06, 0.53) |  | 3.26  (3.05, 3.46) |  | 1.35  (1.20, 1.50) |
| Georgia | 0.06  (0.03, 0.10) | 1.00  (0.55, 1.55) |  | 0.02  (0.00, 0.03) | 0.29  (0.09, 0.60) |  | 0.08  (0.04, 0.13) | 1.38  (0.74, 2.16) |  | 0.03  (0.01, 0.05) | 0.42  (0.12, 0.83) |  | 2.21  (1.56, 2.87) |  | 2.53  (1.84, 3.23) |
| Germany | 2.45  (1.24, 3.94) | 1.91  (0.96, 3.05) |  | 1.76  (0.38, 3.63) | 1.29  (0.28, 2.64) |  | 3.73  (1.98, 5.92) | 1.86  (1.01, 2.95) |  | 2.53  (0.55, 5.01) | 1.10  (0.24, 2.17) |  | -0.44  (-1.02, 0.14) |  | -1.40  (-2.40, -0.39) |
| Ghana | 0.01  (0.00, 0.02) | 0.17  (0.05, 0.35) |  | 0.01  (0.00, 0.02) | 0.21  (0.06, 0.45) |  | 0.11  (0.06, 0.18) | 0.74  (0.40, 1.19) |  | 0.04  (0.01, 0.09) | 0.36  (0.09, 0.73) |  | 5.42  (5.28, 5.57) |  | 1.65  (1.58, 1.72) |
| Greece | 0.16  (0.08, 0.26) | 1.07  (0.51, 1.75) |  | 0.10  (0.02, 0.20) | 0.66  (0.14, 1.36) |  | 0.37  (0.18, 0.59) | 1.44  (0.73, 2.29) |  | 0.24  (0.05, 0.49) | 0.77  (0.17, 1.60) |  | 0.58  (0.29, 0.87) |  | -0.38  (-0.90, 0.14) |
| Greenland | 0.00  (0.00, 0.00) | 2.41  (1.23, 3.85) |  | 0.00  (0.00, 0.00) | 1.43  (0.30, 2.90) |  | 0.00  (0.00, 0.00) | 3.49  (1.84, 5.48) |  | 0.00  (0.00, 0.00) | 1.78  (0.40, 3.63) |  | 1.14  (0.85, 1.44) |  | 0.16  (-0.22, 0.55) |
| Grenada | 0.00  (0.00, 0.00) | 0.64  (0.26, 1.17) |  | 0.00  (0.00, 0.00) | 1.07  (0.28, 2.01) |  | 0.00  (0.00, 0.00) | 1.55  (0.82, 2.48) |  | 0.00  (0.00, 0.00) | 1.69  (0.48, 3.10) |  | 2.96  (2.78, 3.14) |  | 1.62  (1.46, 1.77) |
| Guam | 0.00  (0.00, 0.00) | 1.70  (0.81, 2.82) |  | 0.00  (0.00, 0.00) | 0.95  (0.28, 1.84) |  | 0.00  (0.00, 0.01) | 1.85  (0.97, 2.86) |  | 0.00  (0.00, 0.00) | 0.77  (0.21, 1.46) |  | 0.47  (0.07, 0.87) |  | -0.46  (-0.86, -0.06) |
| Guatemala | 0.01  (0.00, 0.01) | 0.18  (0.05, 0.38) |  | 0.00  (0.00, 0.00) | 0.03  (0.01, 0.12) |  | 0.06  (0.03, 0.11) | 0.58  (0.26, 1.02) |  | 0.01  (0.00, 0.02) | 0.06  (0.02, 0.21) |  | 4.18  (3.75, 4.61) |  | 2.27  (1.82, 2.72) |
| Guinea | 0.00  (0.00, 0.01) | 0.15  (0.05, 0.32) |  | 0.00  (0.00, 0.01) | 0.17  (0.05, 0.36) |  | 0.02  (0.01, 0.03) | 0.32  (0.13, 0.60) |  | 0.01  (0.00, 0.02) | 0.22  (0.06, 0.47) |  | 2.60  (2.57, 2.63) |  | 1.00  (0.96, 1.04) |
| Guinea-Bissau | 0.00  (0.00, 0.00) | 0.22  (0.06, 0.52) |  | 0.00  (0.00, 0.00) | 0.29  (0.07, 0.59) |  | 0.00  (0.00, 0.01) | 0.37  (0.14, 0.72) |  | 0.00  (0.00, 0.00) | 0.34  (0.09, 0.70) |  | 1.62  (1.55, 1.69) |  | 0.60  (0.56, 0.63) |
| Guyana | 0.00  (0.00, 0.00) | 0.68  (0.28, 1.23) |  | 0.00  (0.00, 0.01) | 0.99  (0.27, 1.82) |  | 0.01  (0.00, 0.01) | 1.23  (0.60, 2.09) |  | 0.01  (0.00, 0.01) | 1.28  (0.37, 2.36) |  | 2.11  (1.87, 2.35) |  | 0.77  (0.59, 0.95) |
| Haiti | 0.01  (0.00, 0.02) | 0.24  (0.07, 0.51) |  | 0.02  (0.00, 0.03) | 0.71  (0.18, 1.34) |  | 0.02  (0.01, 0.05) | 0.35  (0.13, 0.72) |  | 0.04  (0.01, 0.09) | 0.82  (0.20, 1.57) |  | 1.68  (1.54, 1.82) |  | 0.65  (0.57, 0.72) |
| Honduras | 0.00  (0.00, 0.01) | 0.21  (0.08, 0.42) |  | 0.00  (0.00, 0.00) | 0.06  (0.02, 0.15) |  | 0.03  (0.01, 0.06) | 0.57  (0.24, 1.07) |  | 0.01  (0.00, 0.02) | 0.13  (0.03, 0.30) |  | 3.59  (3.34, 3.85) |  | 2.47  (2.24, 2.70) |
| Hungary | 0.48  (0.29, 0.71) | 3.29  (1.95, 4.85) |  | 0.12  (0.04, 0.26) | 0.89  (0.28, 1.83) |  | 0.76  (0.45, 1.16) | 3.88  (2.29, 5.92) |  | 0.18  (0.06, 0.38) | 0.87  (0.27, 1.81) |  | 0.40  (0.21, 0.60) |  | -0.37  (-0.70, -0.05) |
| Iceland | 0.00  (0.00, 0.01) | 1.27  (0.63, 2.03) |  | 0.00  (0.00, 0.01) | 1.17  (0.31, 2.21) |  | 0.01  (0.00, 0.01) | 1.31  (0.69, 2.05) |  | 0.01  (0.00, 0.01) | 1.03  (0.26, 1.92) |  | 0.10  (-0.03, 0.22) |  | -0.62  (-0.84, -0.39) |
| India | 0.38  (0.12, 0.81) | 0.09  (0.03, 0.19) |  | 0.86  (0.25, 1.65) | 0.28  (0.09, 0.53) |  | 3.34  (1.63, 5.55) | 0.30  (0.15, 0.51) |  | 3.44  (1.17, 6.49) | 0.38  (0.13, 0.72) |  | 4.17  (3.97, 4.36) |  | 1.38  (1.18, 1.58) |
| Indonesia | 0.15  (0.04, 0.36) | 0.15  (0.04, 0.37) |  | 0.29  (0.06, 0.59) | 0.39  (0.09, 0.79) |  | 1.56  (0.68, 2.84) | 0.70  (0.29, 1.30) |  | 1.34  (0.31, 2.74) | 0.82  (0.19, 1.64) |  | 5.52  (5.44, 5.60) |  | 2.38  (2.25, 2.51) |
| Iran | 0.10  (0.05, 0.18) | 0.42  (0.20, 0.73) |  | 0.13  (0.03, 0.24) | 0.64  (0.20, 1.18) |  | 0.66  (0.39, 0.98) | 0.94  (0.55, 1.40) |  | 0.56  (0.17, 1.01) | 0.88  (0.29, 1.55) |  | 3.04  (2.91, 3.18) |  | 1.25  (1.07, 1.43) |
| Iraq | 0.06  (0.03, 0.10) | 0.76  (0.36, 1.32) |  | 0.05  (0.02, 0.10) | 0.78  (0.27, 1.40) |  | 0.22  (0.12, 0.35) | 1.02  (0.57, 1.57) |  | 0.18  (0.07, 0.31) | 0.94  (0.38, 1.57) |  | 1.36  (1.00, 1.72) |  | 0.97  (0.69, 1.25) |
| Ireland | 0.08  (0.04, 0.14) | 2.07  (1.02, 3.41) |  | 0.10  (0.03, 0.18) | 2.48  (0.78, 4.41) |  | 0.14  (0.08, 0.23) | 1.90  (1.02, 2.99) |  | 0.15  (0.05, 0.26) | 1.92  (0.63, 3.34) |  | -0.49  (-0.56, -0.41) |  | -1.15  (-1.27, -1.02) |
| Israel | 0.08  (0.04, 0.13) | 1.68  (0.83, 2.79) |  | 0.08  (0.02, 0.16) | 1.76  (0.43, 3.34) |  | 0.19  (0.10, 0.30) | 1.58  (0.82, 2.51) |  | 0.18  (0.05, 0.34) | 1.47  (0.39, 2.71) |  | -1.05  (-1.42, -0.68) |  | -1.49  (-1.86, -1.12) |
| Italy | 1.25  (0.62, 2.07) | 1.40  (0.70, 2.32) |  | 1.53  (0.42, 2.85) | 1.69  (0.47, 3.15) |  | 2.13  (1.12, 3.44) | 1.40  (0.73, 2.26) |  | 2.25  (0.65, 4.22) | 1.32  (0.36, 2.47) |  | -0.23  (-0.43, -0.04) |  | -1.24  (-1.48, -1.00) |
| Jamaica | 0.01  (0.00, 0.02) | 0.62  (0.28, 1.08) |  | 0.02  (0.01, 0.03) | 1.01  (0.31, 1.79) |  | 0.05  (0.03, 0.08) | 1.66  (0.92, 2.64) |  | 0.05  (0.02, 0.09) | 1.63  (0.49, 2.94) |  | 3.74  (3.31, 4.17) |  | 1.79  (1.54, 2.05) |
| Japan | 1.10  (0.34, 2.28) | 0.66  (0.20, 1.37) |  | 1.80  (0.34, 3.70) | 1.13  (0.22, 2.27) |  | 2.41  (0.80, 4.88) | 0.65  (0.22, 1.30) |  | 4.66  (1.07, 9.27) | 0.99  (0.21, 2.01) |  | -0.26  (-0.37, -0.16) |  | -0.51  (-0.56, -0.46) |
| Jordan | 0.02  (0.01, 0.03) | 1.24  (0.63, 2.02) |  | 0.01  (0.00, 0.02) | 1.00  (0.32, 1.79) |  | 0.12  (0.07, 0.18) | 2.04  (1.18, 3.02) |  | 0.06  (0.02, 0.11) | 1.28  (0.44, 2.23) |  | 2.07  (1.89, 2.26) |  | 1.21  (1.06, 1.36) |
| Kazakhstan | 0.16  (0.09, 0.25) | 1.28  (0.68, 2.02) |  | 0.06  (0.02, 0.13) | 0.58  (0.17, 1.13) |  | 0.26  (0.15, 0.39) | 1.55  (0.87, 2.32) |  | 0.10  (0.03, 0.19) | 0.67  (0.20, 1.27) |  | 0.88  (0.68, 1.08) |  | 0.59  (0.36, 0.81) |
| Kenya | 0.01  (0.00, 0.03) | 0.16  (0.05, 0.33) |  | 0.00  (0.00, 0.01) | 0.06  (0.02, 0.17) |  | 0.11  (0.06, 0.19) | 0.51  (0.26, 0.86) |  | 0.02  (0.01, 0.05) | 0.09  (0.03, 0.27) |  | 4.68  (4.40, 4.96) |  | 2.12  (1.93, 2.31) |
| Kiribati | 0.00  (0.00, 0.00) | 0.81  (0.35, 1.44) |  | 0.00  (0.00, 0.00) | 0.58  (0.16, 1.09) |  | 0.00  (0.00, 0.00) | 0.83  (0.35, 1.48) |  | 0.00  (0.00, 0.00) | 0.68  (0.19, 1.35) |  | -0.36  (-0.65, -0.08) |  | 0.24  (0.03, 0.45) |
| Kuwait | 0.01  (0.00, 0.01) | 0.85  (0.46, 1.28) |  | 0.00  (0.00, 0.01) | 0.94  (0.39, 1.53) |  | 0.04  (0.02, 0.06) | 1.79  (1.02, 2.67) |  | 0.03  (0.01, 0.05) | 1.36  (0.60, 2.19) |  | 3.72  (3.14, 4.30) |  | 2.22  (1.71, 2.73) |
| Kyrgyzstan | 0.02  (0.01, 0.04) | 0.75  (0.38, 1.24) |  | 0.01  (0.00, 0.02) | 0.29  (0.08, 0.61) |  | 0.03  (0.02, 0.05) | 0.65  (0.34, 1.03) |  | 0.01  (0.00, 0.02) | 0.30  (0.09, 0.60) |  | -0.79  (-1.11, -0.47) |  | 0.32  (0.11, 0.52) |
| Laos | 0.00  (0.00, 0.01) | 0.15  (0.03, 0.40) |  | 0.00  (0.00, 0.01) | 0.25  (0.06, 0.58) |  | 0.03  (0.01, 0.05) | 0.56  (0.24, 1.03) |  | 0.01  (0.00, 0.03) | 0.40  (0.10, 0.85) |  | 4.80  (4.68, 4.93) |  | 1.15  (0.97, 1.33) |
| Latvia | 0.06  (0.03, 0.09) | 1.57  (0.82, 2.44) |  | 0.03  (0.01, 0.06) | 0.88  (0.26, 1.70) |  | 0.08  (0.05, 0.13) | 2.05  (1.13, 3.20) |  | 0.05  (0.02, 0.10) | 1.13  (0.37, 2.04) |  | 0.94  (0.61, 1.28) |  | 1.16  (0.94, 1.37) |
| Lebanon | 0.02  (0.01, 0.04) | 1.08  (0.52, 1.84) |  | 0.03  (0.01, 0.05) | 1.42  (0.53, 2.44) |  | 0.11  (0.06, 0.17) | 2.09  (1.14, 3.31) |  | 0.11  (0.04, 0.19) | 2.05  (0.83, 3.59) |  | 2.98  (2.74, 3.23) |  | 1.79  (1.59, 1.98) |
| Lesotho | 0.00  (0.00, 0.00) | 0.2  4(0.09, 0.49) |  | 0.00  (0.00, 0.00) | 0.20  (0.05, 0.47) |  | 0.01  (0.01, 0.02) | 0.86  (0.42, 1.44) |  | 0.00  (0.00, 0.01) | 0.41  (0.11, 0.84) |  | 4.80  (4.51, 5.09) |  | 2.74  (2.59, 2.88) |
| Liberia | 0.00  (0.00, 0.01) | 0.31  (0.12, 0.60) |  | 0.00  (0.00, 0.01) | 0.29  (0.07, 0.60) |  | 0.01  (0.00, 0.02) | 0.44  (0.19, 0.83) |  | 0.01  (0.00, 0.01) | 0.33  (0.08, 0.71) |  | 2.46  (1.54, 3.38) |  | 1.07  (0.67, 1.47) |
| Libya | 0.02  (0.01, 0.04) | 1.14  (0.54, 2.03) |  | 0.02  (0.01, 0.04) | 1.27  (0.45, 2.35) |  | 0.08  (0.04, 0.13) | 1.64  (0.87, 2.66) |  | 0.06  (0.02, 0.12) | 1.40  (0.48, 2.43) |  | 1.48  (1.24, 1.72) |  | 0.59  (0.41, 0.77) |
| Lithuania | 0.06  (0.03, 0.10) | 1.44  (0.74, 2.32) |  | 0.04  (0.01, 0.07) | 0.87  (0.26, 1.63) |  | 0.12  (0.06, 0.19) | 2.03  (1.07, 3.18) |  | 0.08  (0.02, 0.14) | 1.12  (0.36, 2.02) |  | 1.34  (1.08, 1.61) |  | 1.09  (0.93, 1.24) |
| Luxembourg | 0.01  (0.01, 0.02) | 2.01  (1.05, 3.26) |  | 0.01  (0.00, 0.02) | 1.84  (0.43, 3.64) |  | 0.02  (0.01, 0.02) | 1.46  (0.77, 2.33) |  | 0.01  (0.00, 0.03) | 1.20  (0.30, 2.40) |  | -1.26  (-1.42, -1.10) |  | -1.76  (-1.99, -1.53) |
| Madagascar | 0.01  (0.00, 0.01) | 0.13  (0.04, 0.28) |  | 0.00  (0.00, 0.01) | 0.09  (0.03, 0.22) |  | 0.03  (0.01, 0.05) | 0.25  (0.11, 0.48) |  | 0.01  (0.00, 0.02) | 0.10  (0.03, 0.24) |  | 2.63  (2.33, 2.93) |  | 0.28  (0.20, 0.35) |
| Malawi | 0.00  (0.00, 0.01) | 0.10  (0.02, 0.24) |  | 0.00  (0.00, 0.01) | 0.08  (0.02, 0.18) |  | 0.02  (0.01, 0.04) | 0.30  (0.13, 0.55) |  | 0.01  (0.00, 0.01) | 0.11  (0.03, 0.26) |  | 4.36  (3.94, 4.78) |  | 1.65  (1.50, 1.81) |
| Malaysia | 0.05  (0.02, 0.10) | 0.61  (0.25, 1.16) |  | 0.09  (0.02, 0.17) | 1.15  (0.29, 2.28) |  | 0.38  (0.20, 0.63) | 1.52  (0.80, 2.48) |  | 0.37  (0.10, 0.70) | 1.69  (0.50, 3.13) |  | 2.63  (2.38, 2.88) |  | 0.78  (0.53, 1.03) |
| Maldives | 0.00  (0.00, 0.00) | 0.16  (0.03, 0.42) |  | 0.00  (0.00, 0.00) | 0.54  (0.12, 1.07) |  | 0.00  (0.00, 0.00) | 0.36  (0.16, 0.65) |  | 0.00  (0.00, 0.00) | 0.60  (0.15, 1.18) |  | 2.65  (2.48, 2.81) |  | -0.02  (-0.17, 0.12) |
| Mali | 0.01  (0.00, 0.01) | 0.15  (0.04, 0.33) |  | 0.01  (0.00, 0.01) | 0.17  (0.04, 0.38) |  | 0.03  (0.01, 0.06) | 0.38  (0.16, 0.69) |  | 0.02  (0.00, 0.03) | 0.23  (0.06, 0.50) |  | 3.44  (3.34, 3.55) |  | 0.94  (0.78, 1.10) |
| Malta | 0.00  (0.00, 0.01) | 1.03  (0.48, 1.79) |  | 0.01  (0.00, 0.01) | 2.01  (0.72, 3.39) |  | 0.01  (0.01, 0.02) | 1.20  (0.59, 1.97) |  | 0.02  (0.01, 0.03) | 1.74  (0.65, 2.95) |  | 0.51  (0.22, 0.79) |  | -0.46  (-0.59, -0.32) |
| Marshall Islands | 0.00  (0.00, 0.00) | 0.53  (0.17, 1.03) |  | 0.00  (0.00, 0.00) | 0.77  (0.24, 1.42) |  | 0.00  (0.00, 0.00) | 0.83  (0.34, 1.53) |  | 0.00  (0.00, 0.00) | 0.92  (0.27, 1.71) |  | 1.47  (1.36, 1.58) |  | 0.52  (0.45, 0.60) |
| Mauritania | 0.00  (0.00, 0.01) | 0.45  (0.20, 0.82) |  | 0.01  (0.00, 0.01) | 0.85  (0.28, 1.47) |  | 0.01  (0.01, 0.02) | 0.76  (0.42, 1.23) |  | 0.02  (0.01, 0.03) | 0.91  (0.31, 1.6) |  | 1.77  (1.65, 1.89) |  | 0.20  (0.07, 0.32) |
| Mauritius | 0.00  (0.00, 0.00) | 0.34  (0.16, 0.59) |  | 0.00  (0.00, 0.01) | 0.47  (0.12, 0.93) |  | 0.02  (0.01, 0.03) | 0.99  (0.52, 1.61) |  | 0.01  (0.00, 0.03) | 0.85  (0.22, 1.67) |  | 3.74  (3.45, 4.03) |  | 1.93  (1.70, 2.17) |
| Mexico | 0.22  (0.11, 0.35) | 0.52  (0.26, 0.84) |  | 0.10  (0.02, 0.22) | 0.28  (0.06, 0.59) |  | 1.20  (0.64, 1.86) | 1.03  (0.55, 1.60) |  | 0.47  (0.09, 0.96) | 0.43  (0.09, 0.89) |  | 2.48  (2.41, 2.56) |  | 1.23  (1.03, 1.44) |
| Micronesia | 0.00  (0.00, 0.00) | 0.82  (0.36, 1.45) |  | 0.00  (0.00, 0.00) | 0.82  (0.25, 1.45) |  | 0.00  (0.00, 0.00) | 1.13  (0.52, 1.98) |  | 0.00  (0.00, 0.00) | 1.05  (0.34, 1.90) |  | 0.88  (0.69, 1.08) |  | 0.68  (0.50, 0.85) |
| Moldova | 0.06  (0.03, 0.10) | 1.36  (0.69, 2.23) |  | 0.02  (0.01, 0.04) | 0.51  (0.15, 1.05) |  | 0.12  (0.07, 0.18) | 2.09  (1.20, 3.11) |  | 0.03  (0.01, 0.07) | 0.57  (0.17, 1.13) |  | 2.58  (1.80, 3.36) |  | 1.24  (0.57, 1.92) |
| Monaco | 0.00  (0.00, 0.00) | 2.27  (1.08, 3.73) |  | 0.00  (0.00, 0.00) | 1.99  (0.54, 3.70) |  | 0.00  (0.00, 0.00) | 2.89  (1.41, 4.58) |  | 0.00  (0.00, 0.00) | 2.32  (0.64, 4.19) |  | 0.95  (0.83, 1.07) |  | 0.61  (0.48, 0.73) |
| Mongolia | 0.01  (0.00, 0.01) | 0.53  (0.24, 0.93) |  | 0.00  (0.00, 0.00) | 0.25  (0.07, 0.52) |  | 0.02  (0.01, 0.03) | 0.75  (0.39, 1.24) |  | 0.00  (0.00, 0.01) | 0.33  (0.09, 0.67) |  | 0.76  (0.56, 0.97) |  | 0.66  (0.55, 0.78) |
| Montenegro | 0.01  (0.01, 0.02) | 1.72  (0.95, 2.69) |  | 0.00  (0.00, 0.01) | 0.49  (0.15, 0.97) |  | 0.02  (0.01, 0.04) | 2.47  (1.41, 3.80) |  | 0.01  (0.00, 0.01) | 0.65  (0.19, 1.27) |  | 1.76  (1.57, 1.94) |  | 1.45  (1.30, 1.60) |
| Morocco | 0.05  (0.02, 0.09) | 0.38  (0.18, 0.67) |  | 0.07  (0.02, 0.13) | 0.62  (0.21, 1.07) |  | 0.26  (0.13, 0.43) | 0.86  (0.42, 1.41) |  | 0.25  (0.09, 0.45) | 0.92  (0.35, 1.63) |  | 2.77  (2.54, 3.01) |  | 1.37  (1.17, 1.57) |
| Mozambique | 0.00  (0.00, 0.01) | 0.08  (0.01, 0.20) |  | 0.00  (0.00, 0.01) | 0.07  (0.02, 0.18) |  | 0.03  (0.01, 0.07) | 0.32  (0.12, 0.62) |  | 0.01  (0.00, 0.02) | 0.13  (0.04, 0.30) |  | 5.92  (5.61, 6.23) |  | 2.39  (2.25, 2.53) |
| Myanmar | 0.03  (0.00, 0.07) | 0.11  (0.02, 0.32) |  | 0.05  (0.01, 0.11) | 0.28  (0.06, 0.61) |  | 0.21  (0.08, 0.40) | 0.46  (0.18, 0.87) |  | 0.17  (0.04, 0.35) | 0.46  (0.11, 0.93) |  | 5.64  (5.30, 5.98) |  | 1.89  (1.79, 1.99) |
| Namibia | 0.00  (0.00, 0.00) | 0.26  (0.12, 0.46) |  | 0.00  (0.00, 0.00) | 0.35  (0.09, 0.70) |  | 0.01  (0.00, 0.01) | 0.61  (0.34, 0.99) |  | 0.01  (0.00, 0.01) | 0.61  (0.16, 1.13) |  | 3.10  (2.94, 3.27) |  | 2.00  (1.88, 2.12) |
| Nauru | 0.00  (0.00, 0.00) | 1.62  (0.69, 3.00) |  | 0.00  (0.00, 0.00) | 1.08  (0.28, 2.26) |  | 0.00  (0.00, 0.00) | 1.56  (0.72, 2.66) |  | 0.00  (0.00, 0.00) | 1.08  (0.30, 2.09) |  | -0.71  (-1.15, -0.26) |  | -0.29  (-0.65, 0.07) |
| Nepal | 0.00  (0.00, 0.01) | 0.04  (0.01, 0.11) |  | 0.01  (0.00, 0.02) | 0.16  (0.04, 0.37) |  | 0.04  (0.02, 0.08) | 0.20  (0.08, 0.39) |  | 0.05  (0.01, 0.11) | 0.29  (0.09, 0.61) |  | 5.94  (5.71, 6.17) |  | 2.04  (1.84, 2.25) |
| Netherlands | 0.31  (0.15, 0.52) | 1.54  (0.72, 2.60) |  | 0.15  (0.03, 0.35) | 0.74  (0.15, 1.68) |  | 0.66  (0.32, 1.07) | 1.86  (0.92, 3.04) |  | 0.32  (0.06, 0.71) | 0.85  (0.17, 1.87) |  | 0.63  (0.50, 0.76) |  | 0.43  (-0.06, 0.93) |
| New Zealand | 0.09  (0.05, 0.15) | 2.39  (1.22, 3.78) |  | 0.07  (0.01, 0.14) | 1.78  (0.38, 3.63) |  | 0.18  (0.10, 0.28) | 2.26  (1.22, 3.47) |  | 0.14  (0.03, 0.27) | 1.70  (0.40, 3.20) |  | -0.39  (-0.47, -0.30) |  | 0.02  (-0.18, 0.23) |
| Nicaragua | 0.00  (0.00, 0.01) | 0.30  (0.11, 0.58) |  | 0.00  (0.00, 0.00) | 0.09  (0.02, 0.25) |  | 0.04  (0.02, 0.06) | 0.90  (0.46, 1.49) |  | 0.01  (0.00, 0.02) | 0.22  (0.05, 0.50) |  | 4.06  (3.68, 4.45) |  | 2.98  (2.56, 3.41) |
| Niger | 0.00  (0.00, 0.01) | 0.12  (0.03, 0.26) |  | 0.00  (0.00, 0.01) | 0.19  (0.05, 0.43) |  | 0.01  (0.01, 0.03) | 0.18  (0.07, 0.37) |  | 0.01  (0.00, 0.03) | 0.22  (0.06, 0.48) |  | 1.61  (1.53, 1.69) |  | 0.53  (0.46, 0.60) |
| Nigeria | 0.08  (0.03, 0.17) | 0.19  (0.07, 0.40) |  | 0.09  (0.02, 0.19) | 0.26  (0.07, 0.54) |  | 0.40  (0.19, 0.69) | 0.51  (0.24, 0.89) |  | 0.25  (0.06, 0.51) | 0.39  (0.10, 0.77) |  | 3.73  (3.57, 3.89) |  | 1.72  (1.57, 1.86) |
| Niue | 0.00  (0.00, 0.00) | 1.03  (0.51, 1.71) |  | 0.00  (0.00, 0.00) | 0.86  (0.26, 1.57) |  | 0.00  (0.00, 0.00) | 1.51  (0.81, 2.46) |  | 0.00  (0.00, 0.00) | 1.03  (0.31, 1.93) |  | 1.24  (1.11, 1.38) |  | 0.61  (0.57, 0.66) |
| North Korea | 0.03  (0.01, 0.08) | 0.19  (0.03, 0.49) |  | 0.04  (0.01, 0.09) | 0.38  (0.10, 0.77) |  | 0.07  (0.01, 0.19) | 0.23  (0.04, 0.59) |  | 0.11  (0.03, 0.24) | 0.38  (0.09, 0.79) |  | 0.71  (0.61, 0.80) |  | 0.13  (0.05, 0.21) |
| North Macedonia | 0.03  (0.01, 0.04) | 1.41  (0.73, 2.23) |  | 0.01  (0.00, 0.01) | 0.44  (0.13, 0.90) |  | 0.09  (0.05, 0.14) | 2.78  (1.54, 4.40) |  | 0.02  (0.01, 0.05) | 0.76  (0.23, 1.57) |  | 2.66  (2.38, 2.95) |  | 2.16  (1.86, 2.47) |
| Northern Mariana Islands | 0.00  (0.00, 0.00) | 2.61  (1.45, 4.02) |  | 0.00  (0.00, 0.00) | 1.39  (0.41, 2.55) |  | 0.00  (0.00, 0.00) | 2.58  (1.45, 3.91) |  | 0.00  (0.00, 0.00) | 1.35  (0.39, 2.47) |  | -0.17  (-0.28, -0.07) |  | -0.25  (-0.33, -0.18) |
| Norway | 0.10  (0.05, 0.16) | 1.40  (0.69, 2.33) |  | 0.12  (0.03, 0.24) | 1.66  (0.38, 3.22) |  | 0.15  (0.08, 0.25) | 1.51  (0.77, 2.46) |  | 0.17  (0.04, 0.33) | 1.62  (0.41, 3.10) |  | 0.34  (0.23, 0.46) |  | -0.04  (-0.18, 0.09) |
| Oman | 0.00  (0.00, 0.00) | 0.36  (0.15, 0.71) |  | 0.00  (0.00, 0.01) | 0.79  (0.27, 1.43) |  | 0.02  (0.01, 0.03) | 1.30  (0.72, 1.99) |  | 0.01  (0.00, 0.03) | 1.27  (0.50, 2.16) |  | 4.98  (4.40, 5.56) |  | 2.18  (1.96, 2.39) |
| Pakistan | 0.08  (0.02, 0.19) | 0.15  (0.04, 0.36) |  | 0.18  (0.05, 0.34) | 0.36  (0.11, 0.66) |  | 0.54  (0.25, 0.97) | 0.51  (0.23, 0.92) |  | 0.47  (0.13, 0.86) | 0.55  (0.17, 1.00) |  | 4.57  (4.15, 5.00) |  | 1.34  (1.06, 1.63) |
| Palau | 0.00  (0.00, 0.00) | 0.98  (0.47, 1.67) |  | 0.00  (0.00, 0.00) | 1.08  (0.35, 1.98) |  | 0.00  (0.00, 0.00) | 1.18  (0.61, 1.93) |  | 0.00  (0.00, 0.00) | 1.22  (0.39, 2.17) |  | 0.45  (0.33, 0.57) |  | 0.29  (0.25, 0.33) |
| Palestine | 0.01  (0.00, 0.02) | 0.92  (0.37, 1.83) |  | 0.01  (0.00, 0.02) | 1.44  (0.47, 2.57) |  | 0.04  (0.02, 0.06) | 1.73  (0.88, 2.78) |  | 0.04  (0.01, 0.07) | 2.06  (0.77, 3.48) |  | 2.06  (1.70, 2.42) |  | 1.34  (1.07, 1.61) |
| Panama | 0.01  (0.00, 0.01) | 0.35  (0.10, 0.73) |  | 0.00  (0.00, 0.00) | 0.11  (0.03, 0.27) |  | 0.04  (0.02, 0.07) | 0.92  (0.47, 1.57) |  | 0.01  (0.00, 0.02) | 0.19  (0.05, 0.46) |  | 3.60  (3.51, 3.69) |  | 1.87  (1.77, 1.97) |
| Papua New Guinea | 0.00  (0.00, 0.01) | 0.22  (0.06, 0.51) |  | 0.00  (0.00, 0.01) | 0.34  (0.09, 0.71) |  | 0.02  (0.01, 0.03) | 0.31  (0.10, 0.64) |  | 0.01  (0.00, 0.03) | 0.42  (0.12, 0.83) |  | 0.82  (0.66, 0.98) |  | 0.58  (0.52, 0.64) |
| Paraguay | 0.01  (0.00, 0.01) | 0.39  (0.18, 0.68) |  | 0.00  (0.00, 0.01) | 0.22  (0.04, 0.50) |  | 0.06  (0.03, 0.10) | 1.15  (0.58, 1.91) |  | 0.03  (0.01, 0.06) | 0.55  (0.12, 1.18) |  | 4.07  (3.77, 4.37) |  | 3.51  (3.23, 3.79) |
| Peru | 0.05  (0.02, 0.10) | 0.47  (0.20, 0.83) |  | 0.03  (0.01, 0.06) | 0.25  (0.05, 0.56) |  | 0.29  (0.14, 0.49) | 0.89  (0.43, 1.54) |  | 0.14  (0.03, 0.29) | 0.43  (0.09, 0.91) |  | 2.69  (2.41, 2.96) |  | 2.24  (1.98, 2.50) |
| Philippines | 0.12  (0.05, 0.23) | 0.39  (0.15, 0.75) |  | 0.04  (0.01, 0.11) | 0.20  (0.05, 0.48) |  | 0.63  (0.31, 1.08) | 0.79  (0.38, 1.36) |  | 0.14  (0.04, 0.33) | 0.22  (0.06, 0.52) |  | 2.11  (1.76, 2.46) |  | 0.23  (-0.09, 0.55) |
| Poland | 0.87  (0.48, 1.34) | 2.00  (1.10, 3.07) |  | 0.43  (0.12, 0.85) | 1.03  (0.31, 2.02) |  | 2.34  (1.30, 3.58) | 3.28  (1.83, 5.01) |  | 0.92  (0.28, 1.75) | 1.23  (0.37, 2.35) |  | 1.59  (1.43, 1.76) |  | 0.34  (0.14, 0.54) |
| Portugal | 0.18  (0.08, 0.32) | 1.34  (0.60, 2.34) |  | 0.26  (0.07, 0.48) | 1.94  (0.51, 3.59) |  | 0.45  (0.22, 0.73) | 1.82  (0.89, 2.90) |  | 0.53  (0.15, 0.97) | 1.91  (0.51, 3.48) |  | 0.80  (0.45, 1.15) |  | -0.39  (-0.72, -0.05) |
| Puerto Rico | 0.05  (0.03, 0.08) | 1.38  (0.71, 2.12) |  | 0.04  (0.01, 0.08) | 1.27  (0.41, 2.22) |  | 0.15  (0.08, 0.23) | 2.14  (1.22, 3.36) |  | 0.11  (0.04, 0.20) | 1.41  (0.45, 2.60) |  | 1.56  (1.28, 1.83) |  | 0.37  (0.04, 0.70) |
| Qatar | 0.00  (0.00, 0.00) | 1.54  (0.81, 2.47) |  | 0.00  (0.00, 0.00) | 1.75  (0.68, 3.08) |  | 0.02  (0.01, 0.03) | 3.09  (1.83, 4.59) |  | 0.01  (0.00, 0.02) | 2.20  (0.96, 3.59) |  | 3.20  (2.83, 3.57) |  | 1.45  (1.11, 1.79) |
| Romania | 0.34  (0.20, 0.51) | 1.19  (0.70, 1.82) |  | 0.11  (0.03, 0.23) | 0.43  (0.13, 0.87) |  | 0.98  (0.59, 1.48) | 2.66  (1.60, 3.98) |  | 0.34  (0.10, 0.65) | 0.85  (0.25, 1.60) |  | 2.64  (2.40, 2.87) |  | 2.27  (2.06, 2.48) |
| Russia | 2.43  (1.33, 3.77) | 1.34  (0.73, 2.10) |  | 0.91  (0.27, 1.86) | 0.54  (0.16, 1.09) |  | 4.97  (2.82, 7.41) | 2.10  (1.19, 3.13) |  | 1.75  (0.55, 3.44) | 0.73  (0.23, 1.44) |  | 1.40  (1.11, 1.70) |  | 1.41  (1.13, 1.69) |
| Rwanda | 0.01  (0.00, 0.01) | 0.19  (0.04, 0.45) |  | 0.00  (0.00, 0.01) | 0.11  (0.03, 0.29) |  | 0.02  (0.01, 0.04) | 0.34  (0.13, 0.65) |  | 0.01  (0.00, 0.01) | 0.13  (0.04, 0.30) |  | 2.07  (1.56, 2.58) |  | 0.33  (0.15, 0.50) |
| Saint Kitts and Nevis | 0.00  (0.00, 0.00) | 1.30  (0.64, 2.16) |  | 0.00  (0.00, 0.00) | 1.60  (0.48, 2.91) |  | 0.00  (0.00, 0.00) | 1.86  (0.98, 2.91) |  | 0.00  (0.00, 0.00) | 1.73  (0.51, 3.10) |  | 1.03  (0.85, 1.22) |  | 0.63  (0.47, 0.80) |
| Saint Lucia | 0.00  (0.00, 0.00) | 0.67  (0.29, 1.19) |  | 0.00  (0.00, 0.00) | 1.01  (0.28, 1.87) |  | 0.00  (0.00, 0.00) | 1.09  (0.56, 1.76) |  | 0.00  (0.00, 0.00) | 1.13  (0.35, 2.05) |  | 1.26  (1.05, 1.48) |  | -0.13  (-0.43, 0.17) |
| Saint Vincent and the Grenadines | 0.00  (0.00, 0.00) | 0.54  (0.21, 1.01) |  | 0.00  (0.00, 0.00) | 0.98  (0.27, 1.77) |  | 0.00  (0.00, 0.00) | 1.30  (0.68, 2.09) |  | 0.00  (0.00, 0.00) | 1.33  (0.39, 2.39) |  | 3.07  (2.94, 3.20) |  | 1.13  (0.90, 1.36) |
| Samoa | 0.00  (0.00, 0.00) | 0.97  (0.49, 1.55) |  | 0.00  (0.00, 0.00) | 0.76  (0.24, 1.38) |  | 0.00  (0.00, 0.00) | 0.94  (0.47, 1.53) |  | 0.00  (0.00, 0.00) | 0.79  (0.24, 1.48) |  | -0.37  (-0.48, -0.26) |  | -0.11  (-0.18, -0.03) |
| San Marino | 0.00  (0.00, 0.00) | 2.17  (1.08, 3.57) |  | 0.00  (0.00, 0.00) | 2.00  (0.54, 3.78) |  | 0.00  (0.00, 0.00) | 2.30  (1.04, 4.12) |  | 0.00  (0.00, 0.00) | 1.90  (0.48, 3.84) |  | 0.42  (0.32, 0.52) |  | 0.07  (-0.05, 0.19) |
| Sao Tome and Principe | 0.00  (0.00, 0.00) | 0.44  (0.15, 0.89) |  | 0.00  (0.00, 0.00) | 0.34  (0.08, 0.71) |  | 0.00  (0.00, 0.00) | 1.35  (0.64, 2.48) |  | 0.00  (0.00, 0.00) | 0.59  (0.14, 1.27) |  | 4.12  (4.05, 4.20) |  | 1.94  (1.88, 2.01) |
| Saudi Arabia | 0.03  (0.02, 0.06) | 0.55  (0.26, 0.99) |  | 0.04  (0.02, 0.08) | 0.87  (0.35, 1.54) |  | 0.28  (0.16, 0.42) | 1.56  (0.92, 2.31) |  | 0.22  (0.09, 0.36) | 1.39  (0.64, 2.23) |  | 3.35  (2.98, 3.73) |  | 2.00  (1.74, 2.26) |
| Senegal | 0.01  (0.00, 0.02) | 0.29  (0.11, 0.55) |  | 0.01  (0.00, 0.02) | 0.40  (0.09, 0.79) |  | 0.04  (0.02, 0.06) | 0.51  (0.24, 0.87) |  | 0.03  (0.01, 0.06) | 0.48  (0.12, 0.95) |  | 2.07  (1.87, 2.27) |  | 0.64  (0.52, 0.77) |
| Serbia | 0.24  (0.13, 0.38) | 2.13  (1.11, 3.41) |  | 0.06  (0.02, 0.13) | 0.65  (0.20, 1.28) |  | 0.55  (0.30, 0.86) | 3.41  (1.88, 5.38) |  | 0.14  (0.04, 0.29) | 0.87  (0.25, 1.81) |  | 1.97  (1.80, 2.14) |  | 1.31  (1.14, 1.48) |
| Seychelles | 0.00  (0.00, 0.00) | 0.73  (0.31, 1.35) |  | 0.00  (0.00, 0.00) | 0.70  (0.17, 1.46) |  | 0.00  (0.00, 0.00) | 1.69  (0.87, 2.77) |  | 0.00  (0.00, 0.00) | 1.24  (0.30, 2.51) |  | 2.27  (1.89, 2.65) |  | 1.61  (1.34, 1.88) |
| Sierra Leone | 0.00  (0.00, 0.01) | 0.15  (0.04, 0.35) |  | 0.00  (0.00, 0.01) | 0.22  (0.06, 0.47) |  | 0.01  (0.00, 0.02) | 0.26  (0.10, 0.54) |  | 0.01  (0.00, 0.02) | 0.28  (0.07, 0.58) |  | 2.15  (1.80, 2.49) |  | 0.99  (0.86, 1.12) |
| Singapore | 0.02  (0.01, 0.04) | 0.81  (0.23, 1.72) |  | 0.03  (0.01, 0.05) | 1.50  (0.30, 2.99) |  | 0.07  (0.03, 0.12) | 0.89  (0.44, 1.52) |  | 0.07  (0.02, 0.14) | 1.03  (0.22, 2.02) |  | -0.11  (-0.35, 0.14) |  | -1.57  (-1.70, -1.45) |
| Slovakia | 0.15  (0.08, 0.22) | 2.44  (1.37, 3.73) |  | 0.05  (0.01, 0.09) | 0.79  (0.23, 1.57) |  | 0.32  (0.18, 0.51) | 3.43  (1.90, 5.44) |  | 0.10  (0.03, 0.20) | 1.06  (0.30, 2.18) |  | 1.01  (0.77, 1.25) |  | 1.01  (0.74, 1.30) |
| Slovenia | 0.05  (0.03, 0.08) | 2.15  (1.15, 3.43) |  | 0.02  (0.01, 0.04) | 0.73  (0.22, 1.49) |  | 0.12  (0.07, 0.19) | 2.73  (1.48, 4.23) |  | 0.04  (0.01, 0.08) | 0.83  (0.24, 1.64) |  | 0.72  (0.35, 1.09) |  | 0.45  (0.21, 0.69) |
| Solomon Islands | 0.00  (0.00, 0.00) | 0.42  (0.12, 0.93) |  | 0.00  (0.00, 0.00) | 0.38  (0.10, 0.76) |  | 0.00  (0.00, 0.00) | 0.68  (0.27, 1.27) |  | 0.00  (0.00, 0.00) | 0.54  (0.14, 1.05) |  | 1.36  (1.06, 1.67) |  | 1.04  (0.89, 1.19) |
| Somalia | 0.00  (0.00, 0.01) | 0.08  (0.01, 0.23) |  | 0.00  (0.00, 0.00) | 0.07  (0.02, 0.19) |  | 0.01  (0.00, 0.02) | 0.07  (0.01, 0.25) |  | 0.00  (0.00, 0.01) | 0.07  (0.02, 0.20) |  | 0.12  (-0.01, 0.25) |  | 0.18  (0.07, 0.29) |
| South Africa | 0.17  (0.09, 0.27) | 0.82  (0.43, 1.37) |  | 0.13  (0.04, 0.25) | 0.70  (0.21, 1.38) |  | 0.54  (0.32, 0.80) | 1.26  (0.75, 1.90) |  | 0.29  (0.08, 0.55) | 0.76  (0.22, 1.46) |  | 1.76  (1.51, 2.01) |  | 0.66  (0.25, 1.08) |
| South Korea | 0.11  (0.03, 0.24) | 0.39  (0.11, 0.85) |  | 0.16  (0.03, 0.33) | 0.71  (0.15, 1.44) |  | 0.64  (0.25, 1.18) | 0.72  (0.29, 1.34) |  | 0.82  (0.17, 1.66) | 0.96  (0.21, 1.93) |  | 1.75  (1.44, 2.05) |  | 0.59  (0.37, 0.82) |
| South Sudan | 0.01  (0.00, 0.02) | 0.36  (0.11, 0.84) |  | 0.00  (0.00, 0.01) | 0.14  (0.04, 0.38) |  | 0.02  (0.01, 0.04) | 0.59  (0.26, 1.14) |  | 0.00  (0.00, 0.01) | 0.15  (0.04, 0.38) |  | 2.00  (1.88, 2.12) |  | 0.35  (0.28, 0.42) |
| Spain | 0.84  (0.42, 1.35) | 1.53  (0.76, 2.48) |  | 0.79  (0.18, 1.56) | 1.44  (0.34, 2.80) |  | 2.06  (1.08, 3.22) | 2.02  (1.07, 3.15) |  | 1.83  (0.46, 3.44) | 1.60  (0.38, 3.04) |  | 0.88  (0.69, 1.07) |  | 0.28  (0.19, 0.38) |
| Sri Lanka | 0.01  (0.01, 0.03) | 0.13  (0.05, 0.25) |  | 0.01  (0.00, 0.03) | 0.15  (0.03, 0.32) |  | 0.08  (0.03, 0.13) | 0.31  (0.14, 0.55) |  | 0.04  (0.01, 0.10) | 0.21  (0.05, 0.45) |  | 3.42  (3.29, 3.55) |  | 1.95  (1.76, 2.15) |
| Sudan | 0.02  (0.01, 0.04) | 0.20  (0.08, 0.40) |  | 0.06  (0.02, 0.10) | 0.69  (0.31, 1.25) |  | 0.12  (0.06, 0.24) | 0.69  (0.33, 1.30) |  | 0.17  (0.08, 0.31) | 1.01  (0.45, 1.81) |  | 4.75  (4.49, 5.01) |  | 1.44  (1.34, 1.55) |
| Suriname | 0.00  (0.00, 0.00) | 0.76  (0.34, 1.32) |  | 0.00  (0.00, 0.00) | 1.00  (0.27, 1.83) |  | 0.01  (0.00, 0.01) | 1.48  (0.73, 2.42) |  | 0.01  (0.00, 0.02) | 1.49  (0.46, 2.70) |  | 2.56  (2.27, 2.85) |  | 1.57  (1.30, 1.84) |
| Sweden | 0.19  (0.09, 0.33) | 1.26  (0.60, 2.12) |  | 0.18  (0.04, 0.37) | 1.09  (0.23, 2.21) |  | 0.32  (0.17, 0.51) | 1.45  (0.75, 2.32) |  | 0.23  (0.05, 0.46) | 0.93  (0.20, 1.86) |  | 0.36  (0.20, 0.52) |  | -1.63  (-1.99, -1.28) |
| Switzerland | 0.11  (0.06, 0.18) | 1.06  (0.54, 1.73) |  | 0.12  (0.03, 0.23) | 1.07  (0.25, 2.07) |  | 0.19  (0.09, 0.31) | 1.02  (0.52, 1.65) |  | 0.20  (0.05, 0.37) | 0.98  (0.26, 1.86) |  | -0.40  (-0.55, -0.26) |  | -0.54  (-0.72, -0.35) |
| Syria | 0.02  (0.01, 0.04) | 0.42  (0.20, 0.73) |  | 0.03  (0.01, 0.05) | 0.57  (0.19, 1.01) |  | 0.08  (0.04, 0.13) | 0.68  (0.35, 1.15) |  | 0.07  (0.02, 0.12) | 0.71  (0.26, 1.21) |  | 1.57  (1.31, 1.83) |  | 0.64  (0.41, 0.86) |
| Taiwan (Province of China) | 0.11  (0.05, 0.21) | 0.74  (0.29, 1.34) |  | 0.08  (0.02, 0.16) | 0.65  (0.16, 1.33) |  | 0.69  (0.31, 1.23) | 1.75  (0.78, 3.11) |  | 0.60  (0.16, 1.14) | 1.48  (0.39, 2.83) |  | 3.03  (2.77, 3.29) |  | 2.96  (2.66, 3.25) |
| Tajikistan | 0.01  (0.00, 0.02) | 0.32  (0.11, 0.63) |  | 0.00  (0.00, 0.01) | 0.19  (0.06, 0.40) |  | 0.02  (0.01, 0.04) | 0.46  (0.19, 0.86) |  | 0.01  (0.00, 0.02) | 0.34  (0.10, 0.68) |  | 1.89  (1.08, 2.71) |  | 2.99  (2.55, 3.42) |
| Tanzania | 0.03  (0.01, 0.05) | 0.26  (0.10, 0.52) |  | 0.01  (0.00, 0.02) | 0.11  (0.03, 0.27) |  | 0.13  (0.06, 0.22) | 0.54  (0.26, 0.94) |  | 0.03  (0.01, 0.07) | 0.14  (0.04, 0.36) |  | 2.53  (2.28, 2.78) |  | 1.05  (0.95, 1.15) |
| Thailand | 0.10  (0.03, 0.20) | 0.26  (0.08, 0.56) |  | 0.11  (0.02, 0.23) | 0.41  (0.09, 0.86) |  | 0.66  (0.31, 1.18) | 0.65  (0.31, 1.16) |  | 0.49  (0.12, 1.05) | 0.50  (0.12, 1.07) |  | 2.49  (2.04, 2.94) |  | 0.30  (0.07, 0.52) |
| Timor-Leste | 0.00  (0.00, 0.00) | 0.09  (0.02, 0.24) |  | 0.00  (0.00, 0.00) | 0.26  (0.06, 0.55) |  | 0.00  (0.00, 0.00) | 0.25  (0.07, 0.57) |  | 0.00  (0.00, 0.01) | 0.43  (0.10, 0.91) |  | 4.62  (3.95, 5.29) |  | 2.19  (1.92, 2.47) |
| Togo | 0.00  (0.00, 0.00) | 0.19  (0.06, 0.39) |  | 0.00  (0.00, 0.01) | 0.26  (0.07, 0.54) |  | 0.01  (0.01, 0.02) | 0.38  (0.17, 0.70) |  | 0.01  (0.00, 0.02) | 0.32  (0.08, 0.67) |  | 2.21  (2.10, 2.33) |  | 0.73  (0.69, 0.76) |
| Tokelau | 0.00  (0.00, 0.00) | 0.53  (0.20, 0.98) |  | 0.00  (0.00, 0.00) | 0.63  (0.19, 1.20) |  | 0.00  (0.00, 0.00) | 0.92  (0.44, 1.57) |  | 0.00  (0.00, 0.00) | 0.79  (0.23, 1.49) |  | 1.91  (1.85, 1.97) |  | 0.79  (0.74, 0.85) |
| Tonga | 0.00  (0.00, 0.00) | 0.43  (0.21, 0.72) |  | 0.00  (0.00, 0.00) | 0.43  (0.12, 0.79) |  | 0.00  (0.00, 0.00) | 0.63  (0.34, 1.03) |  | 0.00  (0.00, 0.00) | 0.54  (0.17, 1.00) |  | 0.88  (0.52, 1.24) |  | 0.56  (0.33, 0.79) |
| Trinidad and Tobago | 0.01  (0.01, 0.02) | 1.21  (0.64, 1.93) |  | 0.01  (0.00, 0.02) | 1.51  (0.48, 2.62) |  | 0.03  (0.02, 0.05) | 1.59  (0.85, 2.62) |  | 0.03  (0.01, 0.05) | 1.49  (0.49, 2.66) |  | 1.00  (0.84, 1.16) |  | -0.11  (-0.26, 0.03) |
| Tunisia | 0.03  (0.01, 0.04) | 0.55  (0.26, 0.95) |  | 0.01  (0.00, 0.03) | 0.35  (0.09, 0.72) |  | 0.13  (0.06, 0.21) | 1.03  (0.52, 1.73) |  | 0.06  (0.01, 0.12) | 0.51  (0.13, 1.04) |  | 2.11  (2.04, 2.19) |  | 1.01  (0.84, 1.18) |
| Turkey | 0.42  (0.21, 0.71) | 1.17  (0.59, 1.99) |  | 0.35  (0.10, 0.66) | 1.11  (0.35, 2.07) |  | 1.50  (0.80, 2.31) | 1.72  (0.93, 2.65) |  | 1.08  (0.36, 1.90) | 1.30  (0.44, 2.27) |  | 1.56  (1.04, 2.08) |  | 0.80  (0.36, 1.24) |
| Turkmenistan | 0.01  (0.01, 0.02) | 0.56  (0.29, 0.90) |  | 0.00  (0.00, 0.01) | 0.19  (0.06, 0.38) |  | 0.03  (0.02, 0.05) | 0.80  (0.43, 1.27) |  | 0.01  (0.00, 0.02) | 0.29  (0.09, 0.57) |  | 1.22  (0.65, 1.79) |  | 1.39  (1.07, 1.72) |
| Tuvalu | 0.00  (0.00, 0.00) | 0.48  (0.16, 0.91) |  | 0.00  (0.00, 0.00) | 0.60  (0.16, 1.13) |  | 0.00  (0.00, 0.00) | 0.79  (0.35, 1.43) |  | 0.00  (0.00, 0.00) | 0.72  (0.21, 1.37) |  | 1.38  (1.15, 1.62) |  | 0.39  (0.27, 0.52) |
| Uganda | 0.01  (0.00, 0.02) | 0.14  (0.03, 0.32) |  | 0.00  (0.00, 0.01) | 0.09  (0.03, 0.23) |  | 0.08  (0.03, 0.14) | 0.54  (0.24, 0.97) |  | 0.02  (0.01, 0.04) | 0.16  (0.05, 0.39) |  | 5.35  (5.18, 5.52) |  | 2.16  (1.95, 2.38) |
| UK | 1.91  (0.95, 3.04) | 2.10  (1.05, 3.33) |  | 1.89  (0.48, 3.60) | 1.99  (0.50, 3.77) |  | 2.68  (1.42, 4.10) | 2.05  (1.10, 3.13) |  | 2.32  (0.71, 4.22) | 1.64  (0.48, 3.01) |  | -0.34  (-0.43, -0.26) |  | -0.89  (-1.00, -0.79) |
| Ukraine | 1.44  (0.82, 2.19) | 1.99  (1.14, 3.04) |  | 0.34  (0.10, 0.74) | 0.49  (0.14, 1.04) |  | 1.69  (0.99, 2.58) | 2.22  (1.30, 3.40) |  | 0.34  (0.09, 0.72) | 0.42  (0.12, 0.91) |  | -0.32  (-0.67, 0.03) |  | -1.06  (-1.34, -0.79) |
| United Arab Emirates | 0.01  (0.00, 0.01) | 2.07  (1.01, 3.63) |  | 0.01  (0.00, 0.01) | 2.55  (1.02, 4.62) |  | 0.10  (0.06, 0.15) | 3.36  (1.84, 5.31) |  | 0.05  (0.02, 0.10) | 2.47  (0.99, 4.46) |  | 1.86  (1.44, 2.27) |  | -0.21  (-0.67, 0.26) |
| Uruguay | 0.06  (0.03, 0.11) | 1.67  (0.76, 2.89) |  | 0.03  (0.01, 0.08) | 0.87  (0.16, 1.95) |  | 0.13  (0.07, 0.21) | 2.35  (1.19, 3.75) |  | 0.07  (0.01, 0.16) | 1.21  (0.21, 2.58) |  | 1.03  (0.83, 1.23) |  | 1.14  (0.93, 1.35) |
| USA | 5.74  (2.91, 8.99) | 1.79  (0.92, 2.80) |  | 3.78  (0.91, 7.50) | 1.11  (0.27, 2.21) |  | 10.76  (6.17, 15.58) | 1.93  (1.11, 2.78) |  | 3.53  (0.75, 6.96) | 0.59  (0.13, 1.16) |  | 0.05  (-0.06, 0.15) |  | -2.46  (-2.79, -2.13) |
| Uzbekistan | 0.06  (0.03, 0.09) | 0.48  (0.24, 0.79) |  | 0.02  (0.01, 0.04) | 0.16  (0.05, 0.35) |  | 0.16  (0.09, 0.26) | 0.89  (0.48, 1.43) |  | 0.04  (0.01, 0.08) | 0.41  (0.12, 0.80) |  | 2.08  (1.86, 2.30) |  | 3.82  (3.60, 4.04) |
| Vanuatu | 0.00  (0.00, 0.00) | 0.41  (0.13, 0.83) |  | 0.00  (0.00, 0.00) | 0.29  (0.07, 0.61) |  | 0.00  (0.00, 0.00) | 0.65  (0.28, 1.21) |  | 0.00  (0.00, 0.00) | 0.38  (0.09, 0.75) |  | 1.51  (1.39, 1.63) |  | 0.77  (0.67, 0.88) |
| Venezuela | 0.05  (0.03, 0.09) | 0.57  (0.27, 0.96) |  | 0.01  (0.00, 0.03) | 0.13  (0.03, 0.31) |  | 0.29  (0.14, 0.50) | 1.02  (0.49, 1.73) |  | 0.05  (0.01, 0.12) | 0.20  (0.05, 0.47) |  | 1.68  (1.49, 1.87) |  | 1.19  (1.03, 1.35) |
| Vietnam | 0.04  (0.01, 0.10) | 0.09  (0.02, 0.25) |  | 0.07  (0.02, 0.16) | 0.21  (0.05, 0.44) |  | 0.54  (0.21, 1.02) | 0.58  (0.23, 1.11) |  | 0.34  (0.08, 0.77) | 0.44  (0.11, 1.00) |  | 7.64  (7.30, 7.98) |  | 2.91  (2.76, 3.06) |
| Virgin Islands US | 0.00  (0.00, 0.00) | 1.71  (0.85, 2.77) |  | 0.00  (0.00, 0.00) | 1.18  (0.29, 2.36) |  | 0.01  (0.00, 0.01) | 3.61  (2.03, 5.43) |  | 0.00  (0.00, 0.01) | 1.63  (0.45, 3.12) |  | 3.04  (2.70, 3.38) |  | 1.21  (0.83, 1.59) |
| Yemen | 0.01  (0.00, 0.02) | 0.14  (0.04, 0.32) |  | 0.02  (0.01, 0.04) | 0.51  (0.16, 1.00) |  | 0.04  (0.02, 0.08) | 0.31  (0.14, 0.59) |  | 0.07  (0.02, 0.13) | 0.64  (0.22, 1.22) |  | 3.73  (3.43, 4.04) |  | 1.19  (1.05, 1.33) |
| Zambia | 0.01  (0.00, 0.02) | 0.33  (0.10, 0.70) |  | 0.01  (0.00, 0.01) | 0.27  (0.07, 0.59) |  | 0.05  (0.02, 0.08) | 0.71  (0.32, 1.24) |  | 0.02  (0.00, 0.04) | 0.37  (0.09, 0.77) |  | 2.56  (2.31, 2.82) |  | 1.03  (0.96, 1.10) |
| Zimbabwe | 0.02  (0.01, 0.03) | 0.38  (0.17, 0.70) |  | 0.01  (0.00, 0.01) | 0.20  (0.06, 0.46) |  | 0.04  (0.02, 0.07) | 0.60  (0.28, 1.06) |  | 0.01  (0.00, 0.03) | 0.27  (0.07, 0.62) |  | 1.17  (0.80, 1.56) |  | 0.82  (0.52, 1.11) |

ASMR, age-standardized mortality rate; EAPC, estimated annual percentage change; BMI, body mass index.

## Supplementary Figures

**
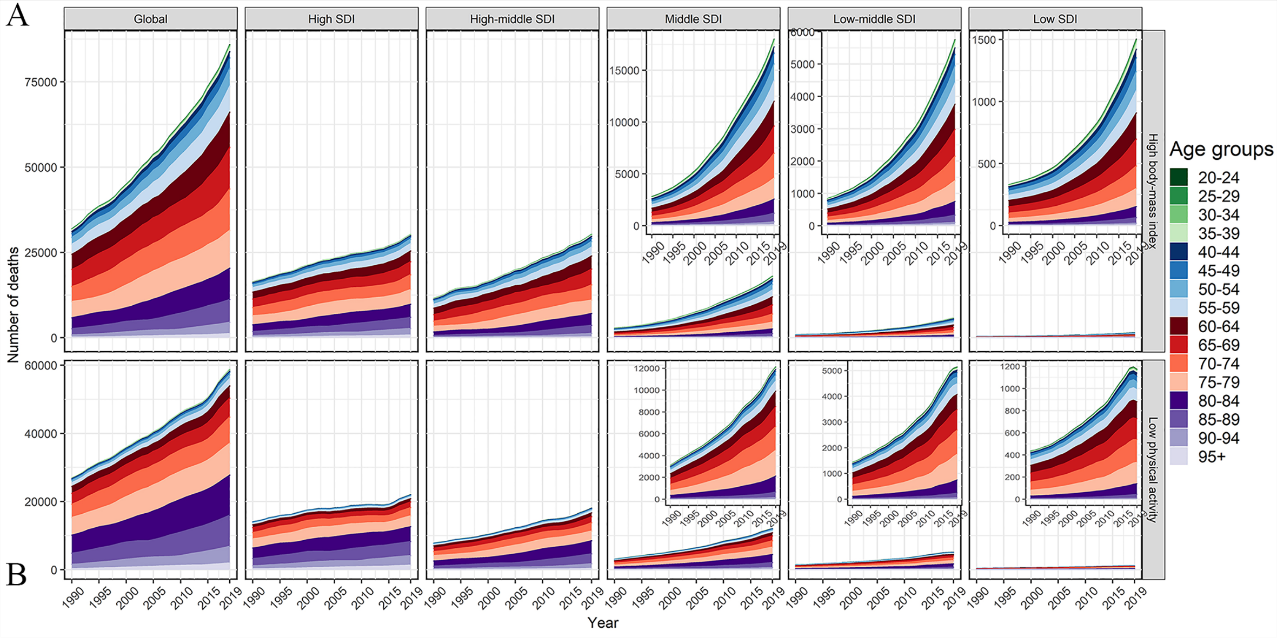
**

**Supplementary Figure 1. The colorectal cancer death numbers due to high BMI and low physical activity by age groups and SDI regions.** (A) high BMI; (B) low physical activity. SDI, sociodemographic index; BMI, body mass index.

**
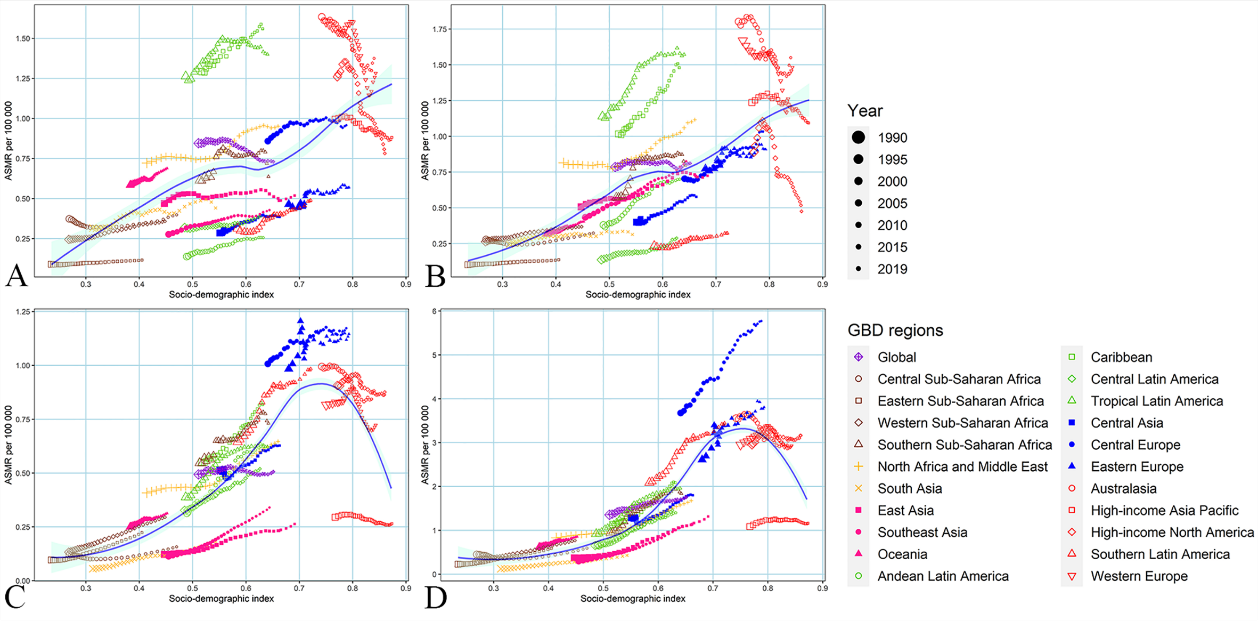
**

**Supplementary Figure 2. The association between low physical activity and high BMI-induced colorectal cancer ASMR with SDI by sex.** (A) low physical activity and female; (B) low physical activity and male; (C) high BMI and female; (D) high BMI and male. ASMR, age-standardized mortality rate; SDI, sociodemographic index; BMI, body mass index.
